# Solution Structures, Dynamics, and Ice Growth Inhibitory Activity of Peptide Fragments Derived from an Antarctic Yeast Protein

**DOI:** 10.1371/journal.pone.0049788

**Published:** 2012-11-28

**Authors:** Syed Hussinien H. Shah, Rajiv K. Kar, Azren A. Asmawi, Mohd Basyaruddin A. Rahman, Abdul Munir A. Murad, Nor M. Mahadi, Mahiran Basri, Raja Noor Zaliha A. Rahman, Abu B. Salleh, Subhrangsu Chatterjee, Bimo A. Tejo, Anirban Bhunia

**Affiliations:** 1 Department of Chemistry, Faculty of Science, Universiti Putra Malaysia, UPM Serdang, Selangor, Malaysia; 2 Department of Biophysics, Bose Institute, Kolkata, West Bengal, India; 3 Malaysia Genome Institute, UKM Bangi, Selangor, Malaysia; 4 Faculty of Biotechnology and Biomolecular Sciences, Universiti Putra Malaysia, UPM Serdang, Selangor, Malaysia; George Washington University, United States of America

## Abstract

Exotic functions of antifreeze proteins (AFP) and antifreeze glycopeptides (AFGP) have recently been attracted with much interest to develop them as commercial products. AFPs and AFGPs inhibit ice crystal growth by lowering the water freezing point without changing the water melting point. Our group isolated the Antarctic yeast *Glaciozyma antarctica* that expresses antifreeze protein to assist it in its survival mechanism at sub-zero temperatures. The protein is unique and novel, indicated by its low sequence homology compared to those of other AFPs. We explore the structure-function relationship of *G. antarctica* AFP using various approaches ranging from protein structure prediction, peptide design and antifreeze activity assays, nuclear magnetic resonance (NMR) studies and molecular dynamics simulation. The predicted secondary structure of *G. antarctica* AFP shows several α-helices, assumed to be responsible for its antifreeze activity. We designed several peptide fragments derived from the amino acid sequences of α-helical regions of the parent AFP and they also showed substantial antifreeze activities, below that of the original AFP. The relationship between peptide structure and activity was explored by NMR spectroscopy and molecular dynamics simulation. NMR results show that the antifreeze activity of the peptides correlates with their helicity and geometrical straightforwardness. Furthermore, molecular dynamics simulation also suggests that the activity of the designed peptides can be explained in terms of the structural rigidity/flexibility, i.e., the most active peptide demonstrates higher structural stability, lower flexibility than that of the other peptides with lower activities, and of lower rigidity. This report represents the first detailed report of downsizing a yeast AFP into its peptide fragments with measurable antifreeze activities.

## Introduction

Sub-zero temperatures are fatal in most organisms by kinetically slowing down vital biochemical reactions, denaturating biomolecules, or rupturing cell membranes. In agreement with Darwin's theory of natural selection, Antarctic and Arctic organisms, including plants, animals, fungi and bacteria, have developed a unique adaptive mechanism of survival by producing antifreeze proteins (AFPs) and antifreeze glycopeptides (AFGPs) [1]. Studies over several decades have revealed that AFPs and AFGPs act as biological inhibitors of ice crystal formation by depressing the water freezing point in a non-colligative manner [2], [3], a process known as thermal hysteresis (TH) [4].

The first AFP was discovered in the blood of Antarctic fish over 40 years ago [5], [6]. Over the past half century, more AFPs have been isolated from different organisms and are now classified into four major types: (1) type I AFPs are described as having Ala-rich protein sequences with amphipathic α-helical structures and varying sizes between 3.3 kDa and 4.5 kDa [7]–[10]; (2) type II AFPs are larger, globular folded proteins with multi-Cys residues bridged by disulphide bonds [11]–[13]; (3) type III AFPs are described as globular proteins with molecular weights of approximately 6 kDa [14]–[17]; and (4) type IV AFPs are α-helical in structure with multi-Glu (E) or Gln (Q) residues in their sequences [18]. In addition, type V AFPs have also been reported from insects and are known as hyperactive proteins [19].

Because of their unique function, AFPs have been proposed to be developed as for commercial products by several reports. For example, some of the current prospects regarding the use of AFPs include extending the expiry date of commercial food products such as frozen meat and yogurt [20], serving as a chemical adjuvant in cryosurgery [21], or supporting the preservation of tissues in transplant [22]. In addition, AFP also has promising utility in genetic engineering where it can be used to increase the cold tolerance of plants and fishes to allow their harvest in cooler climatic conditions [23].

Kun and Mastai [24] hypothesized that smaller antifreeze molecules can act as useful molecular tools for zooming in on the significant portion of antifreeze proteins that contribute to their functionality. Garner and Harding [25] showed that the design of small peptides containing not less than 25 amino acid residues with antifreeze activity is possible. Interestingly, in type I AFPs, it is the α-helical structures of the protein that are responsible for the inhibition of ice crystal growth upon binding to the hydrophobic face of helices with water crystal [26]. Interestingly, not only the α-helical extent of a peptide should be judged but also the composition of the antifreeze peptide should be considered. For instance, though LL37 [27], [28] completely α-helical in nature but it does not have any antifreeze activity. It has also been recently proven that reducing the helical content of AFP by shortening the peptide length results a reduction of the TH value [29]. These facts led us to focus on creating peptide segments with measurable antifreeze activity derived from native AFPs. Attributed to their simpler structure, peptides offer another advantage over large proteins because, in some cases, large protein-based antifreeze molecules do enhance and sustain cold tolerance for a long period of time, whereas in other cases, they do not [30], perhaps due to the complexity of the large protein affecting its stability. Therefore, it can be assumed that peptides may have an advantage over large proteins in terms of their application in areas of medicine, agriculture, and other commercial industries where ice crystal growth is a damaging factor [31].

An Antarctic yeast, *Glaciozyma antarctica* (previously known as *Leucosporidium antarcticum*) [32], that expresses an 18 kDa AFP with very low sequence identity to other AFPs (UniProtKB accession code **D0EKL2**). The sequence dissimilarity of this protein with other AFPs ignited our interest to unlock its structural and functional features, which is the main objective of this paper. The predicted secondary structure of the protein suggests that its α-helical structure is adopted by several small sequences of amino acids. Several peptide fragments were designed based on those sequential strings native to AFP that show α-helical secondary structure. The antifreeze activity of each peptide segment was evaluated by means of thermal hysteresis (TH) and ice recrystallization inhibition (IRI) assays. The peptides show a wide range of measurable antifreeze activities; thus, it has become necessary to correlate the peptide structures with their activities. The ensemble of solution phase structures of the individual antifreeze peptides was determined using NMR spectroscopy at an atomic resolution. The structural straightforwardness and helicity were observed to be the primary factors governing the antifreeze activity of a peptide. The extent of structural helicity in a peptide is proportional to its antifreeze activity.

## Results

### Design of antifreeze peptides

**Figure 1:**
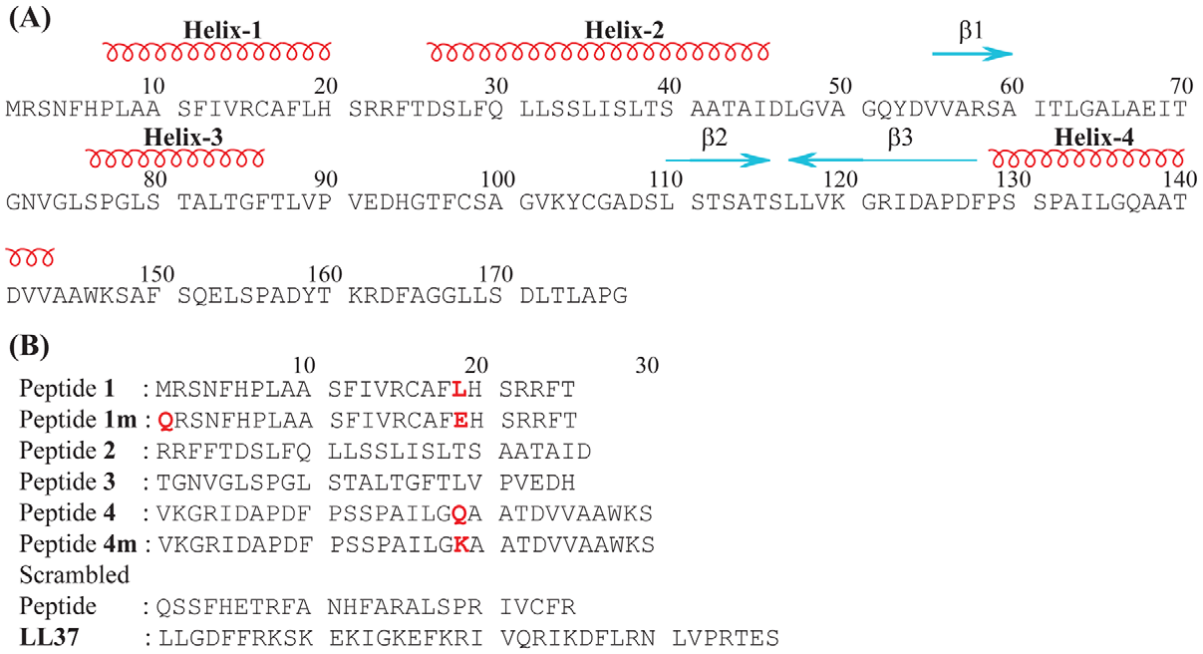
Predicted secondary structure of *G. antarctica* AFP. (A) Primary amino acid sequences of *G. antarctica* AFP. Predicted secondary structure region are shown at the top. (B) *De novo* designed peptide sequences from the α-helical regions of *G. antarctica* AFP. The amino acid mutation in the designed peptide is shown in red.

The yeast *Glaciozyma antarctica* is believed to survive in sub-zero temperature by employing antifreeze protein. This yeast has eight different genes that express various types of AFPs (unpublished data). At the moment, only one AFP gene has been completely characterized (UniProtKB accession code **D0EKL2**). The predicted secondary structure of *G. antarctica* AFP consisting of four α-helices and three β-strands (Figure 1A). The α-helical region of non-glycosylated native AFP has been suggested to be responsible for the inhibition of ice crystal growth by binding the hydrophobic face of the helices to the water crystal [33]. Therefore, the central hypothesis for this study is that the antifreeze activity of *G. antarctica* AFP relies on the α-helical segments of the protein. Small peptides are known to be able to be used as a molecular tool to mimic the biological activity of parent AFPs. As to test our hypothesis, peptide **1** with 25 amino acids in the sequence was designed based on helix-1 of the α-helical regions of *G. antarctica* AFP (Figure 1B). Similarly, peptides **2**, **3** and **4** were also designed mimicking the other three α-helices in the protein (Figure 1B). Peptide **2**, mimicking helix-2 in the protein, is composed of mainly hydrophobic residues and has been found to be insoluble in aqueous medium.

Peptides **1 m** and **4 m** were designed based on the sequence of peptides **1** and **4** by replacing Leu19 with Glu for peptide **1 m** and Gln19 with Lys for peptide **4 m** (Figure 1B). These sequence modifications were performed to assist α-helical structure formation by adding salt bridges in the peptide sequences, which possibly occur at positions *i*, *i+4* or *i*, *i+7* between acidic residues (Asp or Glu) and basic residues (Arg, Lys or His) [25], [34]. Due to these modifications, the modified peptides are expected to form more stable α-helical structure and subsequently have higher antifreeze activity. In addition, the choice of Glu to replace Leu19 in peptide **1 m** was made with careful consideration as the carbon chain length of the Glu side chain is similar to that of Leu (Figure 1B). The same consideration was applied for the replacement of Gln19 with Lys in peptide **4 m** (Figure 1B).

### Evaluations of antifreeze activity

Throughout this work, peptides were solubilized in an unbuffered solution of pH 5.0. The presence of salts in the buffered solution may affect the accuracy of the antifreeze activity assay because saline condition is known to reduce the freezing point [23]. The assay protocol applied in this study is a simple re-crystallization method that enables us to observe ice crystal shape and morphology [35]–[37]. Furthermore, this re-crystallization method also allows us to calculate the TH value of an antifreeze peptide in a solution.

**Figure 2:**
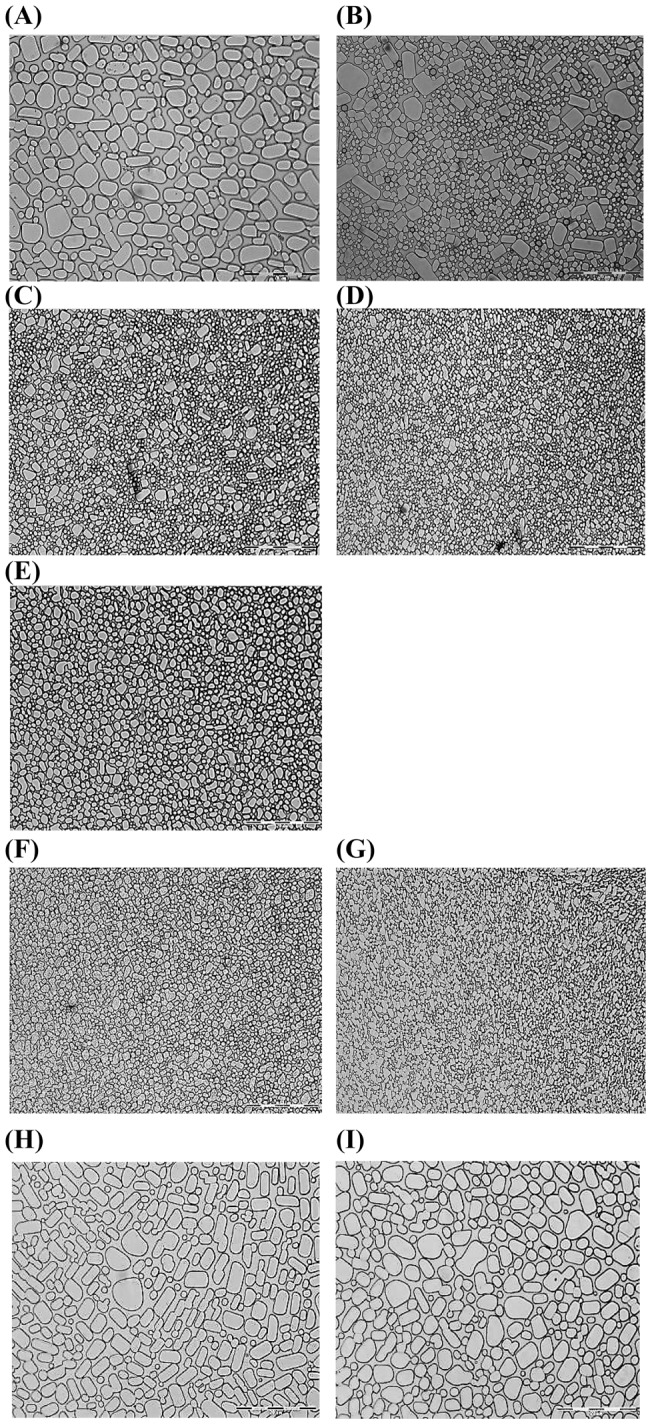
Ice re-crystallization inhibition (IRI) assay. The growth of ice crystal in the presence of either native non-glycosylated protein AFP or 10 mM peptide, that was observed after 3 h of incubation at the temperature of −6°C. (A) Unbuffered water solution (pH 5.0) without peptide, (B) native *G. antarctica* AFP (0.1 mM), (C) peptide **1**, (D) peptide **1 m**, (E) peptide **3**, (F) peptide **4**, (G) peptide **4 m**, (H) scrambled peptide, (I) LL37. The segmented bar represents 100 µm.

The ice recrystallization inhibition (IRI) assay for the peptide in low and high peptide concentrations (1 mM and 10 mM) was observed by measuring the growth of ice crystals after 3 h of incubation at −6°C (Figure 2 and Figure S1) [38]. Recombinant AFP without the signal peptide region from *G. antarctica* expressed in *E. coli* was used as a positive control. A scrambled peptide and a helical peptide, LL37 were used as negative controls. Our results show that native AFP and all five peptides (peptides **1**, **1 m**, **3**, **4**, and **4 m**) did not completely arrest the ice crystal growth but demonstrated slow to moderate growth prior to a well-defined freezing point (Figure 2). These observations indicate that the four peptides (peptides **1**, **1 m**, **4**, and **4 m**) at both low and high concentration can inhibit ice growth better than peptide **3**, which had the lowest IRI activity as marked by the presence of larger ice crystal growth after 3 h of incubation (Figure 2). Interestingly, the mutation of Leu19Glu and Gln19Lys in peptides **1 m** and **4 m**, respectively, showed a clear distinction in the sizes of the ice crystals between the non-modified and modified peptides (Figure 2). The order of antifreeze effectivity on the ice crystal growth was found to be peptide **1 m** > peptide **1**> peptide **4 m** > peptide **4**> peptide **3** amongst all the designed peptides. Both negative controls did not arrest the ice crystal growth (Figure 2).

**Figure 3:**
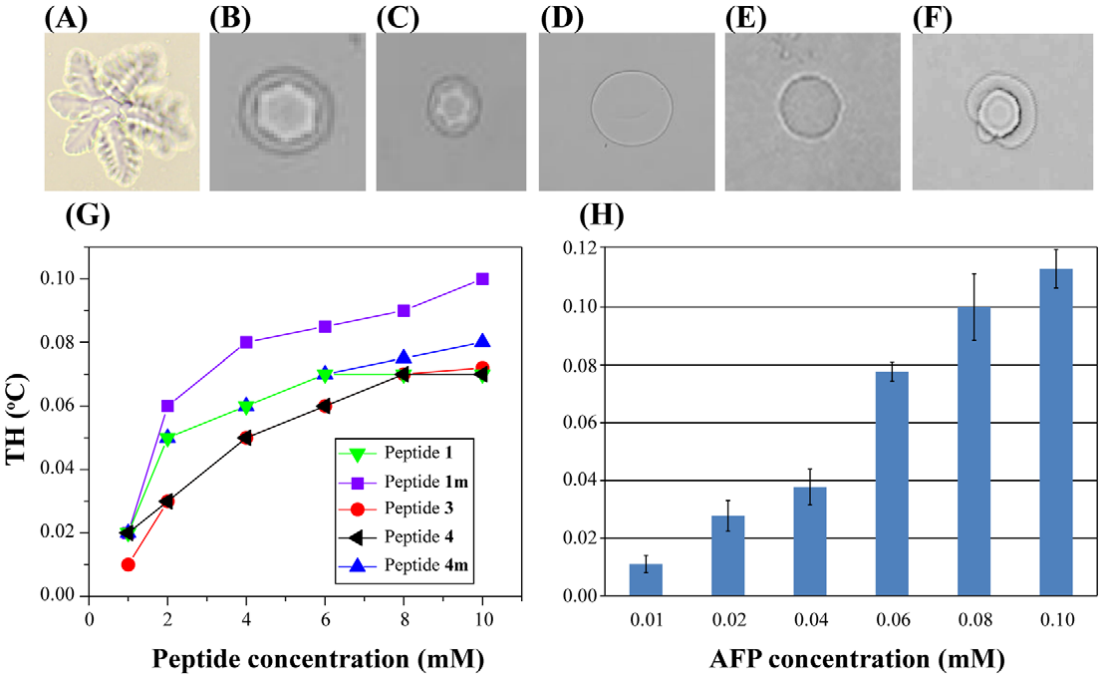
Ice crystal morphology and Thermal hysteresis. Light microscopy images of (A) native *G. antarctica* AFP (0.1 mM), (B) peptide **1 m** (10 mM), (C) peptide **4 m** (10 mM), and (D) unbuffered water solution (pH 5), (E) scrambled peptide, (F) LL37. Thermal hysteresis measurements of (G) peptide **1** (green), **1 m** (purple), **3** (red), **4** (black), and **4 m** (blue) at different concentrations. (H) Thermal hysteresis of native non-glycosylated protein *G. Antarctica* AFP at different concentration.

In addition to lowering the freezing point of water, antifreeze peptides also cause changes in the ice crystal morphology to a hexagonal or similar shape and can completely arrest ice crystal growth at sufficiently high concentrations [39]. The interaction of peptides with the surface of ice crystals and the resulting change in crystal morphology can be studied using simple crystallization experiments. Antifreeze molecules are known to change ice crystal morphology due to their binding to the ice crystal surface. Figure 3A represents the light microscopy image of the positive control (recombinant *G. antarctica* AFP) showing a star-shaped ice crystal at the freezing point. In the presence of peptide **1 m** and peptide **4 m**, a hexagonal shape was formed due to the thermal hysteresis gap, which can be maintained without growing or shrinking between the melting point and the non-equilibrium freezing point of the solution (Figure 3B and 3C). On contrary, unbuffered solution at pH 5.0 without peptide showed zero hysteresis activity with non-restriction of ice crystal growth at 0°C (Figure 3D). It is interesting to observe that both negative controls, scrambled peptide (Figure 1B) and α-helical peptide, LL37 (Figure 1B) do not show any crystal morphology (Figure 3E and 3F). The purpose of using a scrambled peptide as negative control is to prove our hypothesis that the activity of our peptides is sequence-specific; a peptide with random sequence should not have antifreeze activity, which has been successfully proven in our experiment. Another negative control is LL37 peptide, an antimicrobial peptide that has been shown to adopt α-helical structure [27], [28]. This peptide has been taken as the negative control to prove that having helical structure is not sufficient to induce antifreeze activity of the particular peptide. Our result shows that LL37 peptide did not show any measurable antifreeze activity. Therefore, we can conclude that the antifreeze activity of our peptides is specifically correlated with its primary and secondary structures and not a random phenomenon.

In the TH assay, two basic variables are monitored, i.e., peptide concentration and ice crystal growth rate [38]. We tested the antifreeze activity of five peptides at different concentrations, i.e., 1, 2, 4, 6, 8, and 10 mM. The development of single ice crystals was observed in terms of their growth rate at 1°C/minute for slow changes in the ice crystal shape and for the calculation of the TH value. Our results indicate that the antifreeze activity increases as the peptide concentration goes from low to high (Figure 3G). All peptides show measurable hysteresis activity, which is a non-equilibrium phenomenon, by depressing the solution freezing point. Peptide **1 m** at 10 mM concentration shows the highest activity with a TH value of ca. 0.11°C. At this concentration, the activity of peptide **1 m** is higher than that of its non-modified form (peptide **1**), which indicates that the replacement of Leu19 by Glu as Glu19 can stabilize the helicity of peptide **1 m** due to salt bridge or hydrogen bond formation between Arg15 and Glu19. Glu19 can also form a salt bridge or hydrogen bond with Arg23. In a similar fashion, peptide **4 m** shows higher antifreeze activity than its non-modified form (peptide **4**) at 10 mM concentration, which resulted due to the replacement of Gln19 by Lys as Lys19, which was able to stabilize the α-helix by forming a hydrogen bond/salt bridge between Lys19 and Asp23. Peptide **3** shows lower activity at various concentrations compared to peptides **1 m** and **4 m** (Figure 3G). If we consider the sequence of a peptide contributing significantly to its antifreeze activity, then peptide **3** is understood to mainly consist of hydrophobic residues. No inter residual hydrogen bond/salt bridge is expected to form that can stabilize its structure or help it to interact with the ice crystal surface. This reasoning explains why this peptide shows the least affinity to ice crystals and hence was found to have the least antifreeze activity.

Figure 3G is a salient picture (TH vs. peptide concentration) for the assessment of antifreeze activity of the series of peptides in this study. In the TH analysis, the peptide antifreeze activities were as follows: peptide **1 m** > peptide **4 m** > peptide **3**. Peptide **1 m** is the most amphipathic in nature followed by peptide **4 m**, and finally, peptide **3** is mainly hydrophobic in nature. It is interesting to see that the antifreeze activity of a peptide directly connects to the 50% hydrophobicity-50% hydrophilicity nature of a peptide. The amphipathic nature essentially controls its affinity to ice crystals.

The activity of peptides in this study, however, is lower than that of its parent protein. Peptide **1 m** that shows the highest activity among all peptides with TH value of 0.11°C at 10 mM concentration, needs 100 fold higher concentration to reach similar activity with recombinant *G. antarctica* AFP (Figure 3H). An increase in concentration of native protein (>0.1 mM) brings in aggregation and the aggregated protein product gets precipitated. That is why the measurement of antifreeze activity becomes impossible for that protein at higher concentration. The presence of multiple helical regions in the protein structure may explain the higher efficacy of parent protein compared to its derived peptides. Other than helical structures, β-sheet region of the native *G. antarctica* AFP may also contribute to its antifreeze activity. We did not test the latter hypothesis as we only focused on the role of α-helical structure on the activity of *G. antarctica* AFP. Admittedly, the activity of peptides in this study is less superior to that of its parent protein; however, there are several advantages of using peptides compared to their protein parents for future commercial applications. The modular nature of peptides allows for fine-tuning of activity and specificity by replacing single amino acid residue. Since peptides consist of amino acids, they retain the advantages of protein activity and selectivity, but in the same time peptides can be produced in industrial scale with larger quantity than proteins whose production is often marred by sensitivity of biomaterials used during production. We have shown that the activity of peptide **1** can be improved by fine-tuning its structure into peptide **1 m**, which shows some potential that the activity of derived peptides can be improved to match or even better than its parent protein by applying the correct strategy in peptide structural modification.

### NMR studies of antifreeze peptides

**Figure 4:**
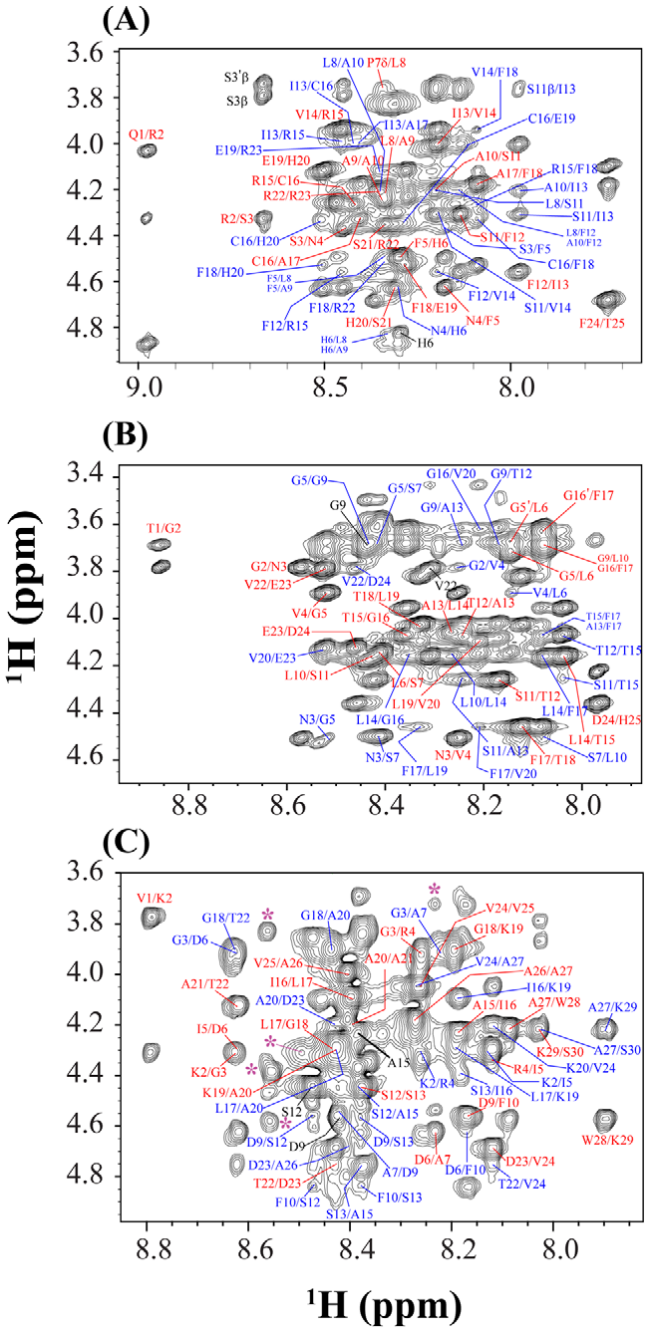
Two-dimensional ^1^H-^1^H NOESY spectra. The finger print region of (A) peptide **1 m**, (B) peptide **3** and (C) peptide **4 m** showing the NOE cross-peaks between C^α^H-HN resonances. The NOESY spectra were acquired with a Bruker 800 MHz spectrometer at 15°C and at a mixing time of 150 ms. Medium range NOEs (C^α^H to NH *i* to *i*+2, *i*+3 and *i*+4) are indicated by blue color, and sequential NOEs (C^α^H to NH *i* to *i*+1) are shown in red. Some peaks (marked by *) are unassigned in the spectrum (C) because of the *cis-trans* configuration of the X-P peptide bond due to presence of Proline residues.

**Figure 5:**
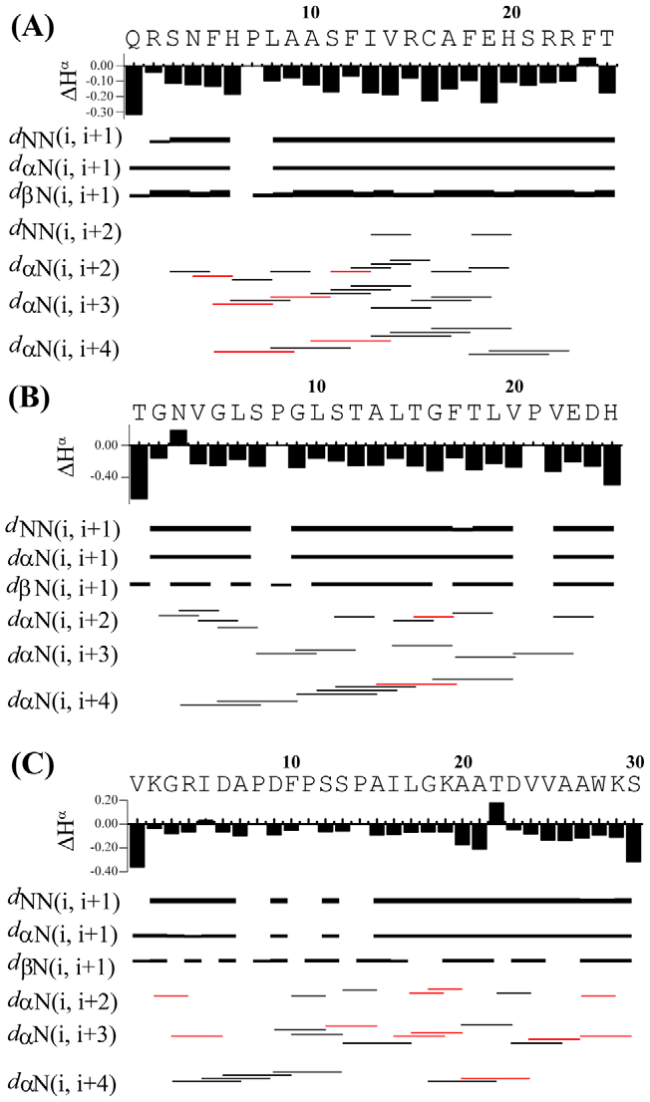
A summary of NMR structural parameters. (A) Bar diagram showing the sequential and medium range NOEs and chemical shift deviations from the random coil values for the C^α^H resonances of peptide **1 m** (A), peptide **3** (B) and peptide **4 m** (C). The thickness of the bar indicates the intensity of the NOESY peaks, which are assigned as strong, medium and weak. Red lines indicate NOE could not be assigned ambiguously due to severe spectral overlap. Amino acid sequences of all the antifreeze peptides are shown at the top.

Peptide **1** and peptide **4** are not considered in this study due to their less potentiality in antifreeze activity compared to that of their mutated analogues, **1 m** and **4 m**. One-dimensional ^1^H NMR spectra of the three peptides (peptides **1 m**, **3**, and **4 m**) show large dispersions of the amide protons (7.7–9.0 ppm), demonstrating that the peptide adopts a folded conformation at low temperatures (Figure S2). These results motivated us to determine the three-dimensional structure of the peptides using NMR spectroscopy. The complete sequence-specific proton resonance assignments of all three peptides were achieved by the analysis of two-dimensional ^1^H-^1^H NOESY and TOCSY spectra [40]. To avoid the severe signal overlap of the NOE cross peaks observed using the Bruker Avance III 500 MHz NMR instrument, the experiment was further carried out using the Bruker DRX 800 MHz NMR magnet. The large numbers of NOE cross-peaks were observed to correlate with the backbone/backbone and backbone/side chain resonances of the three peptides (Figure 4 and S3). For the sequential analysis, the NOESY data unambiguously revealed sequential (C^α^H to NH *i* to *i+1*), medium range (C^α^H to NH *i* to *i+2*, *i+3* and *i+4*) and sequential NH/NH NOEs, a pattern which was ultimately used as a basis for the development of distance restraints to calculate the NMR-derived ensemble of three-dimensional structures (Figure 4 and Figure S3). The data set describing the medium range NOEs completely outlines that all three peptides in the solution phase primarily reside in α-helical conformations (Figures 4 and 5). For peptide **1 m**, it is interesting to see that, from Ser3 to Arg23, almost all of the residues are capable of showing medium range (C^α^H to NH *i* to *i+2*, *i+3* and *i+4*) NOEs, indicating higher order α-helical stability of the middle portion of the peptide (Figure 5). In a similar fashion, the residues Lys2 to Ser30 of peptide **4 m** show medium range (C^α^H to NH *i* to *i+2*, *i+3* and *i+4*) NOEs, defining a well-conserved structure of the central part of the peptide (Figure 5). In addition, due to presence of three Pro residues, Pro8, Pro11 and Pro14, the peptide bond between the residues X-P of peptide **4 m** showed cis/trans isomerization resulting two different sets of resonances (Figure 4). Interestingly, only five residues, namely Ser7, Gly9, Leu14, Phe17 and Val20 in peptide **3** were able to produce *i* to *i+3* NOEs in the two-dimensional ^1^H-^1^H NOESY spectrum (Figures 4 and 5). The numbers of contacts are highest in the case of peptide **1 m** and lowest in the case of peptide **3**, which definitely signifies the higher order stability of peptide **1 m** compared to that of peptide **3**. In peptide **1 m**, from Leu8 to His20, the NOEs range from 12 to 30, this establishes the high order of the structural stability (Figure S4). In peptide **4 m**, the number of NOEs varies from 5 to 16 in a consistent way (Figure S4). However, residues ranging from Gly9 to Thr15 and Phe17 to Val of peptide **3** show a large number of NOE contacts, ranging from 7 to 18 (Figure S4). Most of the residues in peptide **3** and **4 m** failed to produce large number of NOE contacts, indicating higher dynamics of theses peptide in the solution phase (Figure S4).

The chemical shift deviations of the C^α^H resonances from their random coil conformation dictate the extent of secondary conformation of a peptide or protein [41]. The upfield shift of the C^α^H proton confirms the α-helical conformation when a stretch of at least four contiguous residues or a stretch of three adjacent residues showed the same kind of uniform deviation. It is also worthwhile to mention that all three different peptides (peptides **1 m**, **3**, and **4 m**) have α-helical conformation as, for each of them, the ΔH^α^ values of most of the residues were found to be negative (Figure 5).

### Three-dimensional structures of antifreeze peptides

The three-dimensional structures of all three antifreeze peptides were determined based on the distance constraint derived from NOE-based intensities of intra-residual contacts. Among the three, peptide **1 m** is found to be the most stable and have the most uniform α-helical structure. From the ^1^H-^1^H NOESY, the total number of NOEs is found to be 179 (Table 1). Of these, 80 NOEs are found to be sequential and 63 fall into medium range distances. Similarly, for peptide **3**, the value of sequential and medium range NOEs is found to be 69 and 35, respectively. For peptide **4 m**, 24 medium range NOEs and 78 sequential NOEs are used for the structure calculation. The dihedral angles, both φ and ψ, are estimated using the program TALOS.

**Table 1:** Summary of structural statistics for the 20 final structures of three antifreeze peptides.

	Peptide 1m	Peptide 3	Peptide 4m
**Distance restraints**			
Intra-residue (i–j = 0)	35	29	42
Sequential (|i–j| = 1)	80	69	78
Medium-range (2≤|i–j| ≤4)	63	35	24
Long-range (|i–j| ≥5)	01	00	00
Total	179	133	144
**Angular restraints**			
φ	23	22	26
ψ	23	22	26
**Distance restraints from violations (≥ 0.3 Å)**	01	05	01
**Deviation from mean structure (Å)**			
Average back bone to mean structure	0.10±0.06	0.48±0.05	0.33±0.10
Average heavy atom to mean structure	0.93±0.15	0.93±0.18	0.90±0.13
**Ramachandran plot for mean structure**			
% Residues in the most favorable and additionally allowed regions	100	100	100
% Residues in the generously allowed Region	0	0	0
% Residues in the disallowed region	0	0	0

**Figure 6:**
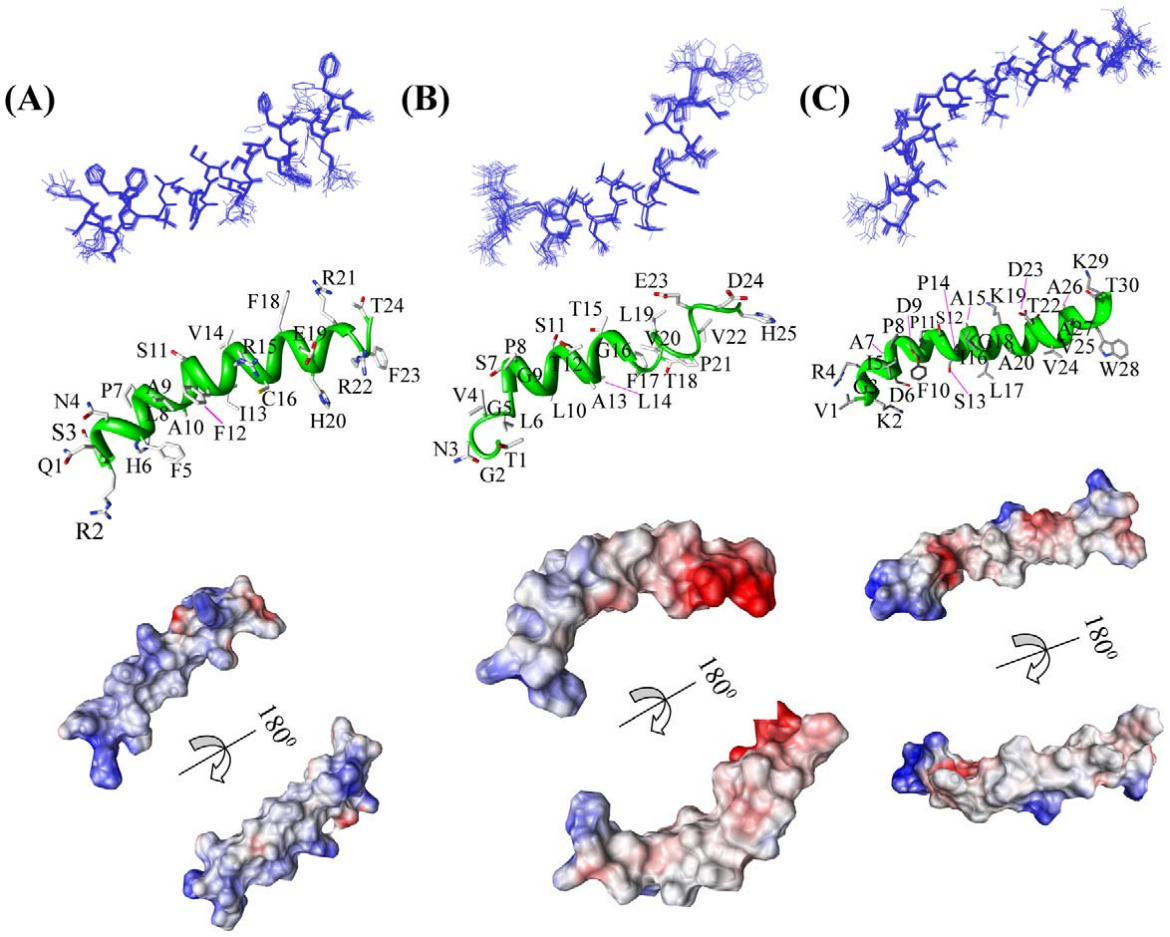
Three dimensional solution structures. Superposition of backbone atoms (N, C^α^ and C') of the twenty lowest energy structures of (A) peptide **1 m**, (B) peptide **3** and (C) peptide **4 m**. Representative helical conformations of the peptides with side chain dispositions are shown for peptide **1 m** (left panel), peptide **3** (middle panel) and peptide **4 m** (right panel). Electrostatic potential surface of peptide **1 m** (left panel), peptide **3** (middle panel) and peptide **4 m** (right panel). The positively charged, neutral and negatively charged amino acid residues are indicated by blue, white and red, respectively. These images were produced using the program Chimera. The atomic coordinates of ensembles of peptides, peptides **1 m**, **3**, and **4 m**, are deposited under PDB accession codes 2LQ0, 2LQ1 and 2LQ2, respectively.

Figure 6 displays the superposition of all backbone atoms (N, C^α^ and C') of the 20 lowest energy structures of peptide **1 m** (Figure 6A), peptide **3** (Figure 6B), and peptide **4 m** (Figure 6C). The three-dimensional solution structures of all three peptides (**1 m**, **3**, and **4 m**) are well defined with average backbone RMSD values of 0.10±0.06 Å, 0.48±0.05 Å, and 0.33±0.10 Å, respectively (Table 1). The RMSD values for the heavy atoms are found to be 0.93±0.15 Å, 0.93±0.18 Å, and 0.90±0.13 Å for peptide **1 m**, peptide **3**, and peptide **4 m**, respectively (Table 1). The solution structure of peptide **1 m** is characterized by an α-helical conformation spanning from Arg2 to Arg22 (Figure 6A, middle panel). However, the long α-helical structure of peptide **1 m** bends at the N-terminal region of the helix, around Phe5-His6-Pro7 (Figure 6A, middle panel). This bent conformation could be due to the combined structural effect induced by the presence of Ser3 and Asn4 consecutively at the N-terminus of the peptide. It is to be noted that these residues exhibit a lower propensity for α-helical structures [42]. Close inspection of the three-dimensional structure of peptide **1 m** clearly indicates that both termini of the peptide are rich in polar residues, whereas the central region is enriched with primarily hydrophobic and aromatic residues. The architecture of peptide **1 m** is tuned in such a way that the terminal ends can be the hands involved in solvating the molecule, whereas the intrinsic straightforward structural pattern of the central part is well maintained by the hydrophobic residual interactions. The additional electrostatic interactions reinforce a strong base in the structural stability of the peptide. In the triad unit Arg15-Glu19-Arg23, *i* to *i+4* side chain/side chain electrostatic interactions govern the structural stability to a huge extent (Figure 6A, middle panel). Interestingly, Phe12-Arg15 and Phe18-Arg22 interact (π-cation type interaction) at the central part of the peptide, maintaining the three-dimensional framework of the peptide (Figure 6A, middle panel). The interesting composition of peptide **1 m** attempts to keep it in a positively charged micro-environment, which keenly helps to solubilize it in the solvent core (Figure 6A, bottom panel). This feature is manifested from the residual electrostatic potential of the peptide (Figure 6A, bottom panel). The adaptive Poisson-Boltzmann surface of charge distribution showed the peptide to be mostly positively charged all across its framework.

In comparison with the straightforward structural pattern of peptide **1 m**, peptide **3** is structurally less straightforward. Although the structure is α-helical, there are two twists in the long helical construction, Pro8 and Thr18, providing an “S” type shape of the peptide (Figure 6B, middle panel). The difference in the structure actually arises due to the sitting of amino acids in the wrong sequence context. The Pro8 governs the first twist assisted by the combined structural neighboring effect of Gly5, Leu6, and Ser7 (Figure 6B, middle panel). In a similar fashion, the deformity at the tail of the C-terminus is brought in by the presence of Thr18. The effect of Thr18 in the structural deformity is imposed by its neighboring residues, such as Thr15, Gly16 and Phe17 which are able to form a β-branched structure (Figure 6B, middle panel) [42]. One very interesting feature in the sequence of peptide **3** is the absence of cationic amino acid residues in the central part of the structure. The structure is basically stabilized by the residual hydrophobic interactions (Figure 6B, middle panel). The electrostatic potential surface demonstrates that this peptide is mainly neutral at its central part, whereas the C- and N-termini are enriched with negative and positive charges, respectively (Figure 6B, bottom panel).

The “L” shaped α-helical structure of peptide **4 m** is due to the presence of three Pro residues, Pro8, Pro11, and Pro14 (Figure 6C, middle panel). The positioning of three consecutive Pro residues is gapped with two amino acids, providing a unique structural element within the long helical framework. This structure creates a kink at the positions of the three Pro residues and thus makes it “L” shaped. Peptide **4 m** is enriched with four positively charged residues, Lys2, Arg4, Lys19, and Lys29, and three negatively charged residues, Asp6, Asp9, and Asp23. The structure is primarily stabilized by the side chain/side chain interactions between hydrophobic residues (Figure 6C, middle panel). The electrostatic potential shows an almost 50–50 arrangement of positive and negative charges throughout the structure (Figure 6C, bottom panel). The atomic coordinates of ensembles of peptides, peptides **1 m**, **3**, and **4 m**, are deposited under PDB accession codes 2LQ0, 2LQ1 and 2LQ2, respectively.

The presence of Trp in a peptide sequence dictates its fluorometric property and also provides structural insight into the peptide. This amino acid individually acts as a probe to understand the flexibility or rigidity of the peptide in the solution state. In peptide **4 m**, Trp28 showed a very high Stern-Volmer constant (Ksv  = 36.55 M^−1^), suggesting that the Trp28 is very dynamic in nature (Figure S5) and does not interact with other neighboring hydrophobic residues. This fact is also reflected by the two-dimensional NOESY spectroscopy. The maximum number of NOEs for Trp28 was found to be 8 (Figure S4). Thus, both fluorescence and NOE data showed Trp28 to be very easily exposed to the solvent.

### Infrared spectroscopy

**Figure 7:**
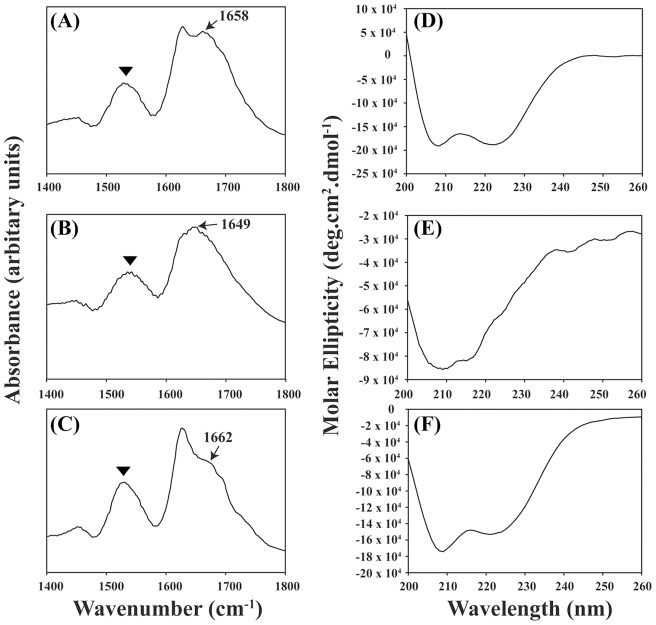
Secondary structure determination using fourier transform IR and CD spectroscopy. α-helical structure of peptide (A) **1 m**, (B) **3** and (C) **4 m** using fourier transform IR spectroscopy. The far UV CD spectra of peptide (D) **1 m**, (E) **3** and (F) **4 m** in water (pH 5.0) with a peptide concentration of ca. 25 µM.

The α-helical nature of all the three peptides comes visible clearly from the FT-IR spectrum where peptide **1 m**, **3** and **4 m** gives characteristic amide **I** and amide **II** vibrational bands. The backbone conformation as related to amide **I** peak is found to be coming at 1658 cm^−1^, 1649 cm^−1^ and 1662 cm^−1^ for peptide **1 m**, **3** and **4 m**, respectively (Figure 7A–C) [43]. Such C = O stretching vibration peaks generally corresponds to α-helix secondary structure. In contrast, amide **II** band is conformationally more sensitive. Unlike peptide **1 m** and **4 m** the absence of a distinct up-rise in the amide **I** peak of peptide **3** might be associated to the two helical twists in the helical construction as because of Pro8 and Thr18. A similar kind of loop is appearing in the amide **II** region at around 1541 cm^−1^ peak. The broadening of the peak for all the three peptides in amide **II** region is attributed to the extended conformations at termini mainly for peptide **1 m** and **4 m**. Such region of amide **II** peaks are shown in highlighted symbols. There is a prominent hump near 1548 cm^−1^ in all the three peptides in the amide **II** region (1510–1580 cm^−1^). Such infrared bands results from the N-H bending vibration (40–60%) and from C-N (18–40%) and C–C stretching vibration (10%). Comprehensively the IR spectrum is in good agreement with the NMR derived secondary structure of peptides.

### CD spectroscopy

Figure 7D–F shows far UV CD spectra of peptide **1 m**, **3** and **4 m**, respectively. The far UV CD spectra of peptide **1 m** and **4 m** is characterized by two negative CD bands, one at ∼ 206 nm and another is at ∼222 nm, which clearly demonstrates that both the peptides exist in alpha helical conformation. However, there is a decrease in the ellipticity values of the diagnostic CD bands of peptide **4 m** in comparison to peptide **1 m**. This observation depicts a partial loss in the helical structure of peptide **4 m**. In contrast, the same CD spectrum of peptide **3** consists of two negative minima, at ∼209 nm and ∼215 nm. The minima at 209 nm is broadened which signifies the large extent of dynamics in the alpha helical content. These fluctuations in the CD band clearly indicate a conformational switch from helical to random coil structure in a dynamic state.

### Molecular dynamics simulation

**Figure 8:**
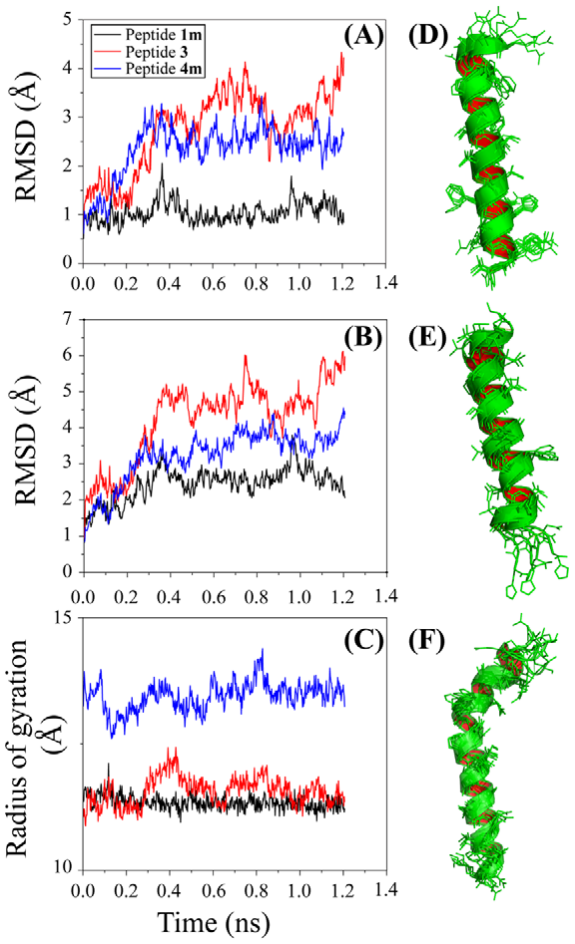
Molecular Dynamics simulation data. (A) A plot of RMSD for back bone vs. time (1.2 ns) for peptide **1 m** (black), peptide **3** (red) and peptide **4 m** (blue). (B) A plot of RMSD for side chain vs. time (1.2 ns) for peptide **1 m** (black), peptide **3** (red) and peptide **4 m** (blue). (C) A plot of RMSD for radius of gyration vs. time (1.2 ns) for peptide **1 m** (black), peptide **3** (red) and peptide **4 m** (blue). Overlay of the snapshots of (D) peptide **1 m**, (E) peptide **3** and (F) peptide **4 m** at 250, 500, 750, 1000, and 1250 ps. These images were produced using the program Chimera.

The NMR-derived solution structures of the antifreeze peptides, peptides **1 m**, **3**, and **4 m**, were uniformly treated in the molecular dynamics simulation at constant temperature and volume to understand their internal dynamics in the water medium. The dynamics simulation is actually a tool to unravel the atomic integrity in a molecular framework. Peptide **1 m** was found to be mainly α-helical and very well packed. The backbone dynamics were found to be very stable in the solvated system, and RMSD values for the backbone for peptide **1 m** were found to be within 1.0 Å from 500 ps to 1200 ps (Figure 8A). The side chain dynamics for peptide **1 m** were also found to be within 1.0±0.6 Å for the same time scale (Figure 8B). The radius for gyration was also calculated and found to vary consistently with RMSD values less than 1.0 Å (Figure 8C). These molecular dynamic features showed that peptide **1 m** is well packed and forms a straightforward, long α-helical structure. The backbone of the peptide is found to be very stable in the water medium. In comparison with the dynamics of peptide **1 m**, peptides **3** and **4 m** demonstrated less stability in the water medium. The backbone dynamics for peptide **3** were found to be very flexible, and RMSD values varied from 2 to 4 Å (Figure 8A). The side chain dynamics for peptide **3** were also found to be very much dynamic in nature, varying the RMSD values from 4 to 6 Å compared to the NMR-derived structures (Figure 8B). Pro8 and Thr18 are the key amino acid residues that affect the α-helical structure of the peptide and create two kinks in the peptide structure. These two residues majorly reinforce the bending of the helicity and generate an “S” shaped structure. The radius of gyration in RMSD for peptide **3** fluctuates widely, meaning that the molecule is very dynamic in nature and from time to time, its helicity is changed (Figure 8C). The backbone dynamics for peptide **4 m** were found to be moderately flexible with RMSD values ranging from 2 to 3 Å (Figure 8A). The side chain dynamics for peptide **4 m** were also found to be moderately dynamic in nature with RMSD values from 2.9 to 4.2 Å compared to the initial NMR-derived structure (Figure 8B). The presence of three repeats of Pro residues (Pro8, Pro11, Pro14) with an interval of two amino acids in a sequence (**Pro**xx**Pro**xx**Pro**) in a *i* to *i+3* manner generates a unique structural element in peptide **4 m**'s structure that alters the long α-helical structure to an “L” shaped one. The radius of gyration in RMSD for peptide **4 m** fluctuates moderately with an RMSD of 13 to 14 Å (Figure 8C). From the dynamics data, it is well understood that peptide **1 m** is very stable in water and forms a straightforward long α-helical structure, whereas peptides **4 m** and **3** are not stable in the water core. The relative water interactions of all three peptides are as follows: peptide **1 m**: water >> peptide **4 m**: water > peptide **3**: water.

The comparisons were found to be well matched with their experimental antifreeze activity assays. The differences in electrostatic iso-surfaces as calculated by APBS are shown diagrammatically in the supplementary material (Figure S6). Figure 8D, 8E, and 8F represent the ensembles of five high resolution structures at the 0.25, 0.5, 0.75, 1.0, and 1.2 ns time scales for peptide **1 m**, peptide **3**, and peptide **4 m**, respectively. The ensemble structures demonstrate that the side chains of peptide **1 m** are well converged, whereas those of peptides **3** and **4 m** are found to be more dynamic (Figure 8D–F).

### Comparative study of the antifreeze activity of peptides from high resolution NMR

**Figure 9:**
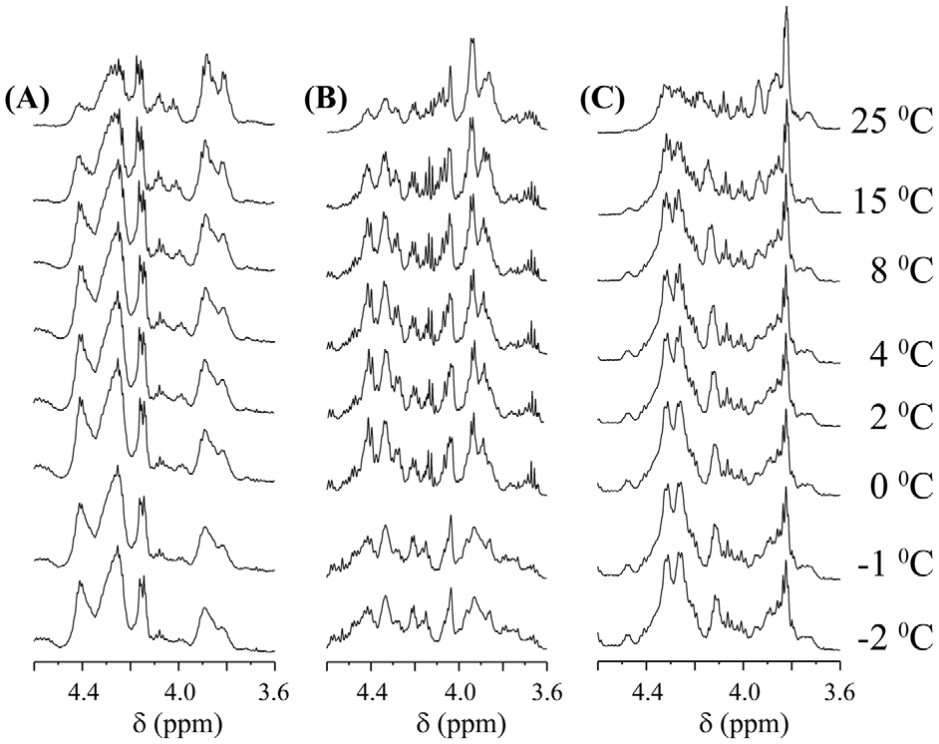
Thermal impact on peptide conformation. One-dimensional ^1^H NMR spectra of (A) peptide **1 m**, (B) peptide **3** and (C) peptide **4 m** using the Bruker Avance 500 MHz instrument. Samples were cooled from 25°C to −2°C as outlined in the Experimental Procedures.

To determine whether there is any change in the structure of three consecutive antifreeze peptides present in normal vs super cooled water, we examined the one-dimensional ^1^H NMR spectra of these peptides. Thus, a series of one-dimensional ^1^H NMR spectra were recorded at various temperatures ranging from 25°C to −2°C (Figure 9) to understand the change of helicity with respect to temperature. The interesting feature in decreasing the temperature is that all of the resonances (aromatic, H^α^, other protons and methyl protons) spanned in the ^1^H NMR spectra are broadened in three consecutive peptides, peptides **1 m**, **3**, and **4 m** (Figure 9). This finding indicates that the three peptides become more α-helical in nature at low temperatures. For clarity, only the H^α^ proton region (3.6 to 4.8 ppm) in the spectrum is selected to monitor the change of helicity of the given peptides. It is worthwhile to mention that the H^α^ resonances are particularly sensitive indicators of protein secondary structure [44]. Broadening of the line width in this region suffices to prove qualitatively that the peptide is interacting with the ice-water surface.

The H^α^ region of peptide **1 m** becomes broader and, at the same time, shifts toward a higher field as the temperature is kept low. This finding signifies that the peptide becomes more α-helical in nature with a lowering the temperature; hence, it is understood that the peptide has more side chain interactions in water near the freezing point. However, interestingly, the broadening of the H^α^ peaks dictates the higher viscosity of the water molecules as the temperature drops. This selectively portrayed more interactions between the peptide and the semi-frozen water. Similarly, the H^α^ resonances of peptide **3** showed a broadening of the peaks as the temperature diminishes. Additionally, the H^α^ resonances shifted toward the higher field with a decrease in the temperature. Broadening of H^α^ resonances depicts a clear-cut picture of increasing interactions of the peptide with semi frozen water. Peptide **4 m** also demonstrated its antifreeze activity by the signature of its proton NMR footprint. Both broadening and changes of the chemical shift of H^α^ resonances are found; hence, it is noted that the structure of peptide **4 m** becomes more α-helical in nature as the temperature decreases. Peptide **4 m** also becomes more prone to interacting with semi frozen water molecules at lower temperatures. A comparative extent of the broadening helped us to categorize the three antifreeze peptides by the efficiency of their antifreeze activities. Broadening is more profound in peptide **1 m**, moderate in peptide **4 m**, and least for peptide **3**. This conclusion from the NMR data in conjunction with other low resolution spectroscopic data matches quite well with the experimental data from the TH assay.

## Discussion

To our knowledge, this work is the first report on the rational design of peptides having measurable antifreeze activity based on the structure of a yeast AFP. There are very few records characterizing the antifreeze activity of bacteria and fungi species. Previously, the psychrophilic fungi *Coprinus psychromorbidus* and *Typhula ishikariensis* have been reported by Hoshino et al. [45], as these species are able to produce unique AFP extra-cellularly. In another work, Gilbert et al. [46] reported the isolation of 866 bacterial isolates from an Antarctic lake, 187 of which showed antifreeze activities. A very recent study by Lee et al. [47] demonstrated that the planar β-sheet structure can be involved in the AFP-ice crystal interaction to suppress the freezing point of ice.

Garner and Harding [25] have conveyed the prospective in a report that the design of small structured peptides with antifreeze activity is possible. The helical structures of AFPs have been suggested as being responsible for the inhibition of ice crystal growth by the binding of the hydrophobic face of the helices to the ice crystal [26]. The length of the peptide also plays an important role, with at least 25 residues being required for antifreeze activity [25]–[26], even though Kun and Mastai [24] reported that shorter peptides (11–13 amino acids) show about 60% of the measurable antifreeze activity of their 37-residue-long parent peptides. Nevertheless, most studies on antifreeze peptides are based on at least 25-residue-long peptides [26], [48]–[50]. All peptides used in this study are 25-residues long, except peptides **4** and **4 m** with 30 amino acids each, with molecular sizes ranging from 2.7 to 3.1 kDa. The peptides used in this study are almost similar in size with other natural antifreeze peptides, such as winter flounder type 1 AFP with a molecular size between 3.3 and 4.5 kDa [7], [14].

The protein isolated from *G. antarctica* is predicted to have a globular shape. The X-ray crystallographic studies failed to elucidate the architecture of the molecule, whereas NMR did not provide any clues or the sub structural insights. The main reason for not elucidating the structure using NMR and X-ray is probably due to the protein's structural elements. It has enormous susceptibility to be aggregated in low concentration in the aqueous medium.

The modifications of peptides **1** and **4** into peptides **1 m** and **4 m** by replacing Leu19 with Glu (peptide **1 m**) and Gln19 with Lys (peptide **4 m**), respectively, were performed with careful considerations of several factors that might improve the activity of an antifreeze peptide. First, it has been shown that helical structure is important for the activity of antifreeze peptides [33], and the stability of the helical structure of the peptides can be enhanced by introducing an *i* to *i+4* lactam bridge plus the N- and C-capping residues [37] or a salt bridge placed on the hydrophilic face of the peptide [34]. Second, the presence of Glu and Leu in peptides **1 m** and **4 m**, respectively, may increase the hydrophilicity of the peptide, which could influence the antifreeze-ice interaction. Several studies have reported that the interaction between antifreeze peptides and water molecules occurs by hydrophilic interaction [25], [26], [31], [36], [39], [51]. Molecular dynamics simulation applied in another study showed that the binding of the hydrophilic surface of the peptide to water molecules provides a layer of unstructured water molecules that stops ice crystals from growing further [52]. Another advantage of having more acidic or basic amino acids in the peptide sequence is to minimize the challenges during the synthesis process and also to overcome the solubility problem of the peptide in water.

**Figure 10:**
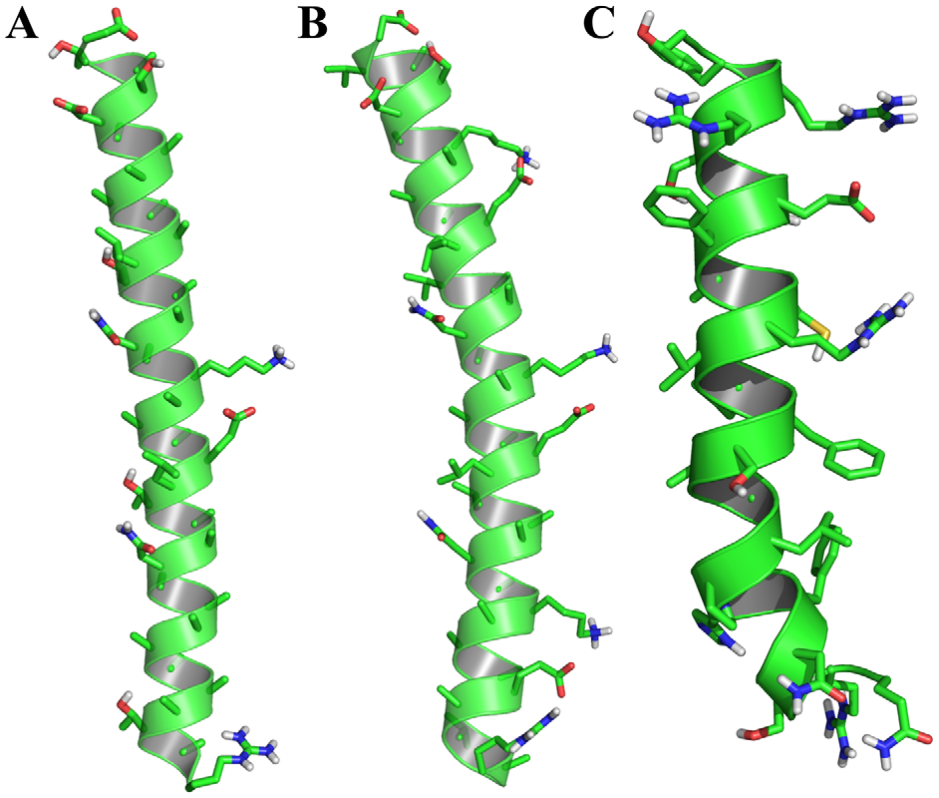
Comparison of type I antifreeze peptide structures. The three-dimensional structures of (A) winter flounder AFP (1WFB.pdb), (B) mutated winter flounder AFP (1J5B.pdb), and (C) peptide **1 m** (2LQ0.pdb). These images were produced using the program PyMOL.

To determine the structure-activity correlation for the designed antifreeze peptides, we determined the three-dimensional structure of peptides **1 m**, **3**, and **4 m** in solution using high resolution NMR spectroscopy (Figure 6). It is noteworthy to mention that the curved structures of these antifreeze peptides are very common in nature, in particular antimicrobial peptides and peptides in the membrane exhibit this type of structure [53]–[55]. The importance of helix straightforwardness was very well demonstrated earlier, particularly in AFP type I [56], [57]. The geometrical straightforwardness (Figure 10) helps in stabilizing the peptide on ice crystal plane. The straightforwardness of the peptide **1 m** derived from the high resolution NMR is quite similar to that of the X-ray crystal structures of winter flounder AFP (1WFB.pdb) (Figure 10A) and its mutated analogue (1J5B.pdb) (Figure 10B). It has been suggested that AFP type I undergoes an equilibrium between straight and bent helices in solution, combined with independent equilibrium between different side chain rotamers on some of the amino acid residues [57].

The mystery of the mechanism of the antifreeze activity of this type of peptide is under investigation. To unravel the underlying mechanism of antifreeze activity, a significant adsorption inhibition mechanism was proposed. Antifreeze proteins are cited as binding irreversibly to ice rather than migrating with the ice-water interface. Further crystal growth is restricted to the free, unblocked surface between the adsorbed, surface-bound ‘impurities’ and leads to an increase in the curvature of the ice-water interface in these regions [17]. Knight et al. showed that AFP can bind with the oxygen atom of a primary plane of ice by Thr and Asp side chains, donating hydrogen bonds [58]. Wen and Laursen investigated hydrogen bonding between AFP and the ice surface and found that Thr and Asp side chains bound to the oxygen atoms of ice water on the {20–21} plane of ice in the <01–12> direction [59]. In contrast, the electric dipole of a polypeptide was proposed to play a substantial role in determining its antifreeze activity [60]. When an AFP is aligned on the ice surface, it attempts to make an electrical macro-dipole where the N terminal is positively charged and the C terminal is negatively charge. The potential difference due to the existence of the charge reinforces the proper alignment of the peptide on the ice surface. The assembly of AFP on the ice surface is actually dictated by the interactions of the macro-dipole of the AFP and the dipoles of the water molecules in the surface. Taken together, five main factors govern the antifreeze activity of AFPs. They play very crucial roles in inhibiting the ice crystal growth in water, as follows: (i) Ala-rich AFPs induce highly helical conformations such that they can sit very well on the ice crystal surface, (ii) macro-dipoles induced by the AFPs inhibit crystal growth, (iii) amphiphilicity of the helix, (iv) the presence of charged polar residues, and (v) torsional freedom of the side chains in the AFP facilitate hydrogen bonding to the ice surface.

**Figure 11:**
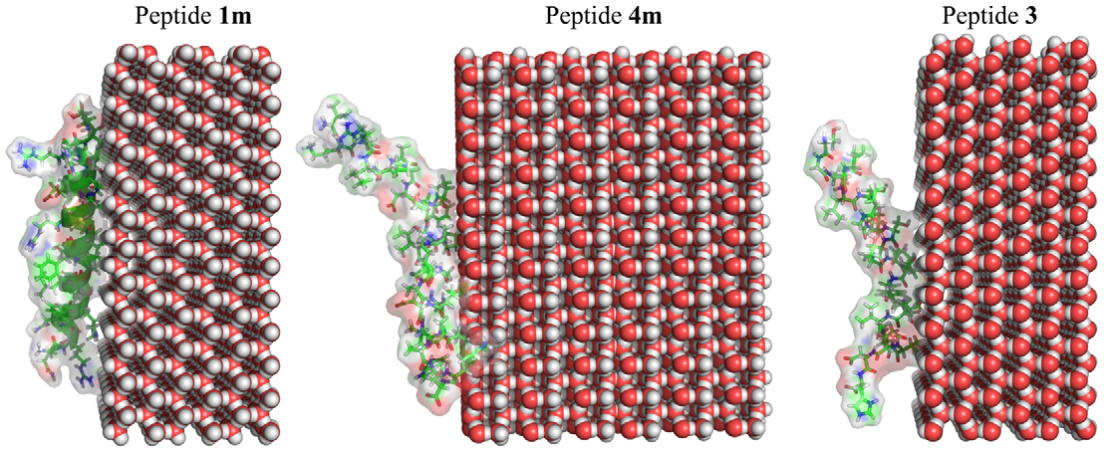
Model of peptide structures at the ice surface. The structure of peptide **1 m** shows a straightforward α-helix and sits on the ice surface (left panel) better than that of peptide **4 m**, which adopts an “L”-shaped α-helical structure (middle panel). Due to its “S”-shaped α-helical structure, peptide **3** shows weaker interaction with the ice surface (right panel) than that of peptides **1 m** and **4 m**. This Figure suggests a structure-function relationship of antifreeze peptide, which shows that higher degree of geometrical straightforwardness correlates with higher antifreeze activity. These images were produced using the program PyMOL.

Figure 11 describes the efficacy of peptides **1 m**, **3**, and **4 m** attaching to the ice crystal surface. It has been already shown from the IRI assay (Figure 2) that peptide **1 m** inhibits ice crystal formation more efficiently compared to peptides **3** and **4 m**. From the NMR-derived structure and MD simulation and also CD spectroscopy, it has been clear that peptide **1 m** retains a straightforward, long α-helical structure. Peptide **1 m** is found to be the least dynamic among the three peptides. It is observed that the hydrophobic residues, particularly Thr, Leu, Ser, and Ile are directly involved in the interaction with the ice crystal surface. The structure of peptide **3** is very dynamic in nature and adopts an “S” shape in solution. Whereas peptide **4 m** retains an α-helical conformation, it's dynamics fall between those of peptides **1 m** and **3**. IRI, as well as the MD simulation of NMR-derived structures of the peptides, confirm as peptide **1 m** is the least dynamic and straightforward α-helical in nature, it can sit on the ice surface very efficiently (Figure 11). In contrast, peptide **3** with “S” shape and varied dynamics cannot be accommodated on the ice surface for long time. The structure and dynamics of peptide **4 m** are found to be in the middle of those of peptides **1 m** and **3**. The “L” shaped structure of peptide **4 m** does not allow it to interact with the ice surface in a significant manner, thus reducing its antifreeze activity.

Here, we used the knowledge of sequence dependence on the structure of the peptides. We observed that Pro and Gly residues remarkably change the α-helical structure, reinforcing the antifreeze activity. Discarding Pro and Gly residues from the peptide sequence can probably deliver well-conserved α-helical structures of the peptides, which can have significant antifreeze activity. Our hypothesis is that the antifreeze activities of a small peptide situated in different environments, alone or placed within a protein, is preserved. The methodology is unique. We are in the process of gaining knowledge of structure-sequence context relationships that may lead us to discover novel antifreeze peptides in near future for industrial and medical use.

## Materials and Methods

All the peptides (**1**, **1 m**, **2**, **3**, **4**, **4 m, scrambled** and **LL37**) studied in this project were purchased from GL Biochem, Shanghai, China, with 98% purity. The molecular weights of these peptides were confirmed by ESI mass spectrometry.

### Secondary structure of G. antarctica antifreeze protein

The sequence of *G. antarctica* AFP consisting of 177 amino acids was taken from UniProtKB (accession code **D0EKL2**). PSI-BLAST [61], [62] and CLUSTALW [63] analyses showed low percentages of sequence identity (less than 30%) between *G. antarctica* AFP and other antifreeze proteins that rendered homology modeling based protein structure prediction impossible. The secondary structure of *G. antarctica* AFP was predicted using a mixture of hyper threading and *ab initio* computational methods [64], [65]. The uniqueness of this protein was confirmed by the fold recognition/threading method based on a similarity search in the secondary structure of the query protein and known proteins in the Protein Data Bank. Three methods were used for the similarity search: mGenThreader [66], 3DPSSM [67], and FUGUE [68], and none of these three methods showed any significant fold similarity between *G. antarctica* AFP and any protein in the Protein Data Bank.

### Peptide design

The peptides were designed based on the amino acid sequence of α-helical regions in the predicted structure of *G. antarctica* AFP. Four peptides were derived from the sequence of *G. antarctica* AFP without modification (peptide **1**–**4**), whereas two peptides (peptide **1 m** and **4 m**) were peptides **1** and **4**, respectively, with residual modifications expected to form additional salt bridges and result in an enhancement of the propensity of stable α-helix formation. The sequence of peptide **1 m** was derived from peptide **1** with Leu19Glu replacement to allow the formation of *i*, *i+4* or *i*, *i-4* helix-stabilizing salt bridges with neighboring residues Arg15 or Arg23. Another modification was Met1 to Gln to avoid the possibility of Met oxidation, thus potentially increasing peptide stability. Peptide **4 m** was a modified form of peptide **4** with a Gln19Lys replacement to allow the formation of an *i*, *i+4* salt bridge with residue Asp23. Peptide **2** was insoluble in water due to its hydrophobicity. Therefore, this peptide was dropped from all experiments. The sequences of all peptides used in this study are listed in Figure 1.

### Ice recrystallization inhibition (IRI) assay

All peptide samples were prepared in unbuffered solution (pH 5.0) and were then diluted with 30% sucrose in a 1∶1 ratio. A volume of 1.5 µL of diluted sample was sandwiched between two 13 mm diameter circular glass cover slips. The sandwiched sample was cooled to −70°C using the programming unit and then maintained at −6°C for 3 h [38]. After the incubation period, ice crystals were observed under a light microscope attached to a temperature control machine. Finally, a comparison of the recrystallization of ice was made by comparing to control samples (30% sucrose without AFP). The same protocol was applied to the positive control, which was native *G. antarctica* AFP expressed in *E. coli* system. Two peptides were used as the negative controls: (i) a scrambled peptide derived from peptide **1 m** with systematic scrambling to nullify the presence of any α-helix-strengthening salt bridges, and (ii) an antimicrobial peptide (LL37) that has been shown to adopt α-helical structure [27].

### Thermal hysteresis (TH) assay

The synthesized peptides, were dissolved in an aqueous solution of pH 5.0 at different concentrations, i.e., 1, 2, 4, 6, 8 and 10 mM. The positive control was native G. antarctica AFP (0.1 mM). The TH assay was carried out by dropping 1 μL of peptide solution onto a glass slide and placing it at the center of the temperature controller system (THM 600S, Linkam Scientific Instruments, Surrey, UK). The peptide-containing sample was heated to 20°C and then chilled to −40°C at a rate of 100°C/min. The sample was heated again to −5°C at 100°C/min, and then the heating rate was decreased to 1°C/min until one single ice crystal was formed with a diameter of approximately 10 µm, which was appropriate to observe the modification of ice crystal shapes. The temperature was then decreased slowly (at 1°C/min) to observe the ice crystal growth. The TH value was calculated by subtracting the temperature at which a single ice crystal ice formed from the temperature at which the ice crystal stopped growing. Ice crystal morphology changes were observed and recorded using computer software (cellˆD, Olympus, Hamburg, Germany) connected to the microscope. The same protocol was applied to the positive control.

### Crystallography study

The effects of antifreeze peptides on the crystal morphology and crystallization kinetics were determined by cryomicroscopy based on a previous method [24]. A drop (1 μL) of the peptide solutions or positive control solution was frozen on a microscope slide with an average cooling rate of 1°C/min, from room temperature (20°C/min) to −5°C/min, before the temperature was adjusted until a single ice crystal obtained at 1°C on a cooling stage (THM 600S, Linkam Scientific Instruments, Surrey, UK) under a microscope (Olympus BX51, Olympus, Hamburg, Germany).

### NMR experiments

Two-dimensional NMR experiments were carried out on both Bruker Avance/DRX spectrometer operating at 800 MHz (Biomolecular NMR Laboratory, University of Kansas, USA) and 500 MHz (Bose Institute, Kolkata, India). The peptides were dissolved in 600 µL of 90% H_2_O: 10% D_2_O, and the pH was adjusted to 5.0. Data collection was accomplished using the time proportional phase incrementation (TPPI) method at 15°C. Two-dimensional TOCSY (mixing time, 80 ms) and Nuclear Overhauser Effect Spectroscopy (NOESY) experiments were carried out for the sequential assignment and structure calculation, respectively. Three different mixing times, 150, 200, and 250 ms, were used for the NOESY experiments. The NOESY experiment was performed with 456 increments in *t1* and 2K data points in *t2* using excitation sculpting for residual water suppression [69]. The spectral width was normally 12 ppm in both dimensions. After 16 dummy scans, 80 scans were recorded per *t1* increment. After zero-filling in t1, 4K (*t2*) × 1K (*t1*) data matrices were obtained. All ^1^H chemical shifts were referenced using DSS (2,2-dimethyl-2-silapentane-5-sulfonate sodium salt) as an internal standard (0 ppm). The two-dimensional NMR data were processed by TopSpin software suite (Bruker, Switzerland) and analyzed using the program SPARKY [70]. The interaction of the peptide with the solvent water was examined by recording a series of one-dimensional proton NMR spectra using Bruker Avance III 500 MHz spectrometer with decreasing the temperature from 25°C to −2°C.

### Peptide structure determination

The NOE cross-peak intensities from the two-dimensional NOESY spectra acquired with a mixing time of 150 ms were classified qualitatively as strong, medium, and weak, which was then translated to upper bound distance limits of 2.8, 3.5, and 5.0 Å, respectively. The lower bound distance was restricted to 2.0 Å to avoid van der Waals repulsion. In addition, backbone dihedral angle (φ and ψ) restraints were derived from TALOS [71] using H^α^ chemical shifts of the designed peptides [72]. These predicted dihedral angle constraints were used for structure calculation with a variation of ±20° from the average values. The DYANA program (version 1.5) [73] was used for structure calculation. However, to refine the structure, several rounds of structure calculation were carried out based on the NOE violations, and the distance constraints were adjusted accordingly. A total of 100 structures were calculated, and 20 conformers with the lowest energy values were selected to present the NMR ensemble. The stereo chemical quality of the structures was determined using the program PROCHECK-NMR [74].

### Infrared spectroscopy

FT-IR spectra for all three peptides, Peptide **1 m**, **3** and **4 m** were recorded on a Fourier transform infrared spectrometer FTIR-8400S (Shimadzu FTIR Spectrometer) at room temperature. The samples are analyzed in the single beam optical system using Germanium-coated KBr plate as beam splitter equipped with temperature controlled high sensitivity detector (DLATGS detector). The powder form of peptides were mixed with KBr powder, placed in a sample cup and then processed for spectrum measurement (diffuse reflectance method) at a resolution of 4 cm^−1^. Data sampling in the instrument was done with He-Ne laser. The diffuse reflected spectra were converted into transmission spectra for comparison purpose using Kubelka-Munk conversion spectra in the IR software [75]. The concept of Fourier self deconvolution is based on the assumption, that a spectrum of single bands is broadened in the liquid or solid state. The deconvoluted spectrum was fitted with Gaussian band shapes by an iterative curve fitting procedure.

### Circular Dichroism

Secondary structure of peptides, **1 m**, **3** and **4 m** were determined using far UV CD method. Peptides were dissolved in water (pH 4.5). CD data were collected using a Jasco-715 spectropolarimeter. The far UV CD spectra were obtained over a range of 200–260 nm using a quartz cell of 2.0 mm path length at 10°C. For each analysis, three scans were accumulated and averaged. All CD spectra were corrected by subtraction of baseline. The corrected CD data obtained in millidegree (θ) were converted to molar ellipticity in deg.cm^2^.dmol^−1^.

### Molecular dynamics simulation of NMR-derived structures of antifreeze peptides

The molecular dynamics simulation for all antifreeze peptides was carried out using CHARMM version-34b1 [76]. The standard CHARMM protein and nucleic acid residue topology and parameter files were used [77]. The initial energy minimization for all peptides was accomplished using 500 steepest descent steps followed by Adopted Basis Newton-Rhapson steps up to their convergence. Each system was solvated using a TIP3P [78] water model with a 12 Å distance between the box edge and the peptides, and the peptide-water system was neutralized using counter ions as required. Water molecules closer than 2.8 Å from any atom of solute or counter ions were deleted. The solvated system was then subjected to energy-minimization, keeping the solute fixed, for 1000 Adopted Basis Newton-Raphson steps, to allow the water molecules and counter ions to orient themselves around the solute. All three systems were processed for a cycle of heating (100 K to 300 K) – constant temperature (300 K) – cooling (300 K to 100 K) for 20 ps each phase. The cycle was repeated three times. The method was adapted with a vision to achieve favourable global minima for all three peptides. Before the production run for dynamics simulation, each system was again heated up to 300 K for 20 ps and then equilibrated at constant temperature and pressure for the next 40 ps so as to provide sufficient time to distribute the velocity equally among all atoms. Hence, in this way, the above process proceeded for a time scale of 240 ps. SHAKE [79] was applied with a tolerance of 1.0e-10 to constrain the bonds involving hydrogen atoms, enabling a 2 fs integration time step to be used. Molecular dynamics simulation at a constant temperature and volume was carried out with the help of Newton's equation of motion using the Leap Verlet algorithm. Particle Mesh Ewald (PME) [80] was used to handle electrostatic interactions. A nonbonded cutoff of 16 Å was used, and the coordinates were saved after each 1 ps. The NMR-derived structures of each peptide underwent molecular dynamics simulation of a 1.2 ns time scale. The structures at the end of the simulation, i.e., the last 300 ps, were found to be convergent within 1 to 1.5 Å rmsd deviations. The electrostatic iso-surfaces of the final structures for each peptide after finishing the molecular dynamics simulation were compared with their NMR-derived initial structures. Adaptive Poisson Boltzmann Solver [81] was used to calculate the iso-surfaces with a range of −15 K/eT to +15 K/eT.

### Theoretical model for ice crystal-peptide interaction

The structural models of molecular interactions between ice crystal and peptides **1 m**, **3**, and **4 m** were built using rigid docking computational method implemented in HEX 6.3 program [82]. The energy minimization was performed to remove the steric constraints and the lowest energy conformations were selected.

## Supporting Information

Figure S1 Ice re-crystallization inhibition (IRI) assay results. The growth of ice crystal in the presence of 1 mM peptide was observed for 3 h at the temperature of −6°C. (A) Unbuffered water solution (pH 5.0) without peptide, (B) peptide **1**, (C) peptide **1 m**, (D) peptide **3**, (E) peptide **4**, (F) peptide **4 m**. The segmented bar represents 100 µm.
(TIF)

Figure S2 Low field region of the one-dimensional proton NMR spectra of peptide **1 m** (top), peptide **3** (middle) and peptide **4 m** (below).
(TIF)

Figure S3 Selected region of two-dimensional ^1^H-^1^H NOESY spectra of peptide **1 m** (left panel) and peptide **3** (right panel).
(TIF)

Figure S4 Bar diagram showing the NOE contacts for each residue of peptide **1 m**, peptide **3,** and peptide **4 m**.
(TIF)

Figure S5 Fluorescence quenching of peptide **4 m** by acrylamide in water, pH 5.0. The Stern-Volmer constant (Ksv) for the peptide is 36.55 M^−1^.
(TIF)

Figure S6 APBS calculation per residues of peptide **1 m** (A) NMR structure and (B) after 1.2 ns MD; Peptide **3** (C) NMR structure and (D) after 1.2 ns MD; Peptide **4 m** (E) NMR structure and (F) after 1.2 ns MD. Blue color indicates the negatively charged amino acid residue whereas the positively charged residues are marked by blue color.
(TIF)

## References

[1] Davies PL, Sykes BD (1997) Antifreeze proteins. Curr Opin Struct Biol 7: 828–834. 9434903 10.1016/s0959-440x(97)80154-6

[2] Duman JG, Olsen TM (1993) Thermal hysteresis protein activity in bacteria, fungi, and phylogenetically diverse plants. Cryobiology 30: 322–328.

[3] Ewart KV, Lin Q, Hew CL (1999) Structure, function and evolution of antifreeze proteins. Cell Mol Life Sci 55: 271–283. 10188586 10.1007/s000180050289 PMC11146970

[4] Jia Z, Davies PL (2002) Antifreeze proteins: an unusual receptor-ligand interaction. Trends Biochem Sci 27: 101–106. 11852248 10.1016/s0968-0004(01)02028-x

[5] Scholander PF, van Dam L, Kanwisher JW, Hammel HT, Gordon MS (1957) Supercooling and osmoregulation in arctic fish. J Cell Compar Physl 49: 5–24.

[6] DeVries AL (1971) Glycoproteins as biological antifreeze agents in antarctic fishes. Science 172: 1152–1155. 5574522 10.1126/science.172.3988.1152

[7] Duman JG, Devries AL (1974) Freezing resistance in winter flounder Pseudopleuronectes americanus. Nature 247: 237–238.

[8] Duman JG, de Vries AL (1976) Isolation, characterization, and physical properties of protein antifreezes from the winter flounder, Pseudopleuronectes americanus. Comp Biochem Physiol B 54: 375–380. 1277804 10.1016/0305-0491(76)90260-1

[9] Hew CL, Joshi S, Wang NC, Kao MH, Ananthanarayanan VS (1985) Structures of shorthorn sculpin antifreeze polypeptides. Eur J Biochem 151: 167–172. 4029130 10.1111/j.1432-1033.1985.tb09081.x

[10] Chao H, Hodges RS, Kay CM, Gauthier SY, Davies PL (1996) A natural variant of type I antifreeze protein with four ice-binding repeats is a particularly potent antifreeze. Protein Sci 5: 1150–1156. 8762146 10.1002/pro.5560050617 PMC2143429

[11] Slaughter D, Fletcher GL, Ananthanarayanan VS, Hew CL (1981) Antifreeze proteins from the sea raven, Hemitripterus americanus. Further evidence for diversity among fish polypeptide antifreezes. J Biol Chem 256: 2022–2026. 6780556

[12] Ng NF, Trinh KY, Hew CL (1986) Structure of an antifreeze polypeptide precursor from the sea raven, Hemitripterus americanus. J Biol Chem 261: 15690–15695. 3782083

[13] Ng NF, Hew CL (1992) Structure of an antifreeze polypeptide from the sea raven. Disulfide bonds and similarity to lectin-binding proteins. J Biol Chem 267: 16069–16075. 1644794

[14] Chao H, Davies PL, Sykes BD, Sonnichsen FD (1993) Use of proline mutants to help solve the NMR solution structure of type III antifreeze protein. Protein Sci 2: 1411–1428. 8401227 10.1002/pro.5560020906 PMC2142453

[15] Jia Z, DeLuca CI, Davies PL (1995) Crystallization and preliminary X-ray crystallographic studies on Type III antifreeze protein. Protein Sci 4: 1236–1238. 7549887 10.1002/pro.5560040621 PMC2143154

[16] DeLuca CI, Chao H, Sonnichsen FD, Sykes BD, Davies PL (1996) Effect of type III antifreeze protein dilution and mutation on the growth inhibition of ice. Biophys J 71: 2346–2355. 8913575 10.1016/S0006-3495(96)79476-6 PMC1233724

[17] Sonnichsen FD, DeLuca CI, Davies PL, Sykes BD (1996) Refined solution structure of type III antifreeze protein: hydrophobic groups may be involved in the energetics of the protein-ice interaction. Structure 4: 1325–1337. 8939756 10.1016/s0969-2126(96)00140-2

[18] Deng G, Andrews DW, Laursen RA (1997) Amino acid sequence of a new type of antifreeze protein, from the longhorn sculpin Myoxocephalus octodecimspinosis. FEBS Lett 402: 17–20. 9013849 10.1016/s0014-5793(96)01466-4

[19] Liou YC, Davies PL, Jia Z (2000) Crystallization and preliminary X-ray analysis of insect antifreeze protein from the beetle Tenebrio molitor. Acta Crystallogr D Biol Crystallogr 56: 354–356. 10713525 10.1107/s0907444999016844

[20] Griffith M, Ewart KV (1995) Antifreeze proteins and their potential use in frozen foods. Biotechnol Adv 13: 375–402. 14536093 10.1016/0734-9750(95)02001-j

[21] Koushafar H, Pham L, Lee C, Rubinsky B (1997) Chemical adjuvant cryosurgery with antifreeze proteins. J Surg Oncol 66: 114–121. 9354167 10.1002/(sici)1096-9098(199710)66:2<114::aid-jso8>3.0.co;2-g

[22] Chao H, Davies PL, Carpenter JF (1996) Effects of antifreeze proteins on red blood cell survival during cryopreservation. J Exp Biol 199: 2071–2076. 8831147 10.1242/jeb.199.9.2071

[23] Fan Y, Liu B, Wang H, Wang S, Wang J (2002) Cloning of an antifreeze protein gene from carrot and its influence on cold tolerance in transgenic tobacco plants. Plant Cell Rep 21: 296–301.

[24] Kun H, Mastai Y (2007) Activity of short segments of Type I antifreeze protein. Biopolymers 88: 807–814. 17868093 10.1002/bip.20844

[25] Garner J, Harding MM (2007) Design and synthesis of alpha-helical peptides and mimetics. Org Biomol Chem 5: 3577–3585. 17971985 10.1039/b710425a

[26] Harding MM, Ward LG, Haymet AD (1999) Type I ‘antifreeze’ proteins. Structure-activity studies and mechanisms of ice growth inhibition. Eur J Biochem 264: 653–665. 10491111 10.1046/j.1432-1327.1999.00617.x

[27] Durr UHN, Sudheendra US, Ramamoorthy A (2006) LL-37 the only human member of the cathelicidin family of antimicrobial peptides. Biochim Biophys Acta 1758: 1408–1425. 16716248 10.1016/j.bbamem.2006.03.030

[28] Ramamoorthy A (2009) Beyond NMR spectra of antimicrobial peptides: Dynamical images at atomic resolution and functional insights. Solid State Nucl Magn Reson. 35: 201–207. 10.1016/j.ssnmr.2009.03.003 PMC2694728 19386477

[29] Park KS, Jung WS, Kim HJ, Shin SY (2010) Determination of the minimal sequence required for antifreeze activity of type I antifreeze protein (AFP 37). Bull Korean Chem Soc 31: 3791–3793.

[30] Barrett J (2001) Thermal hysteresis proteins. Int J Biochem Cell Biol 33: 105–117. 11240367 10.1016/s1357-2725(00)00083-2

[31] Garner J, Harding MM (2010) Design and synthesis of antifreeze glycoproteins and mimics. Chembiochem 11: 2489–2498. 21108270 10.1002/cbic.201000509

[32] Turchetti B, Thomas HallSR, Connell LB, Branda E, Buzzini P, et al (2011) Psychrophilic yeasts from Antarctica and European glaciers: description of Glaciozyma gen. nov., Glaciozyma martinii sp. nov. and Glaciozyma watsonii sp. nov. Extremophiles 15: 573–586. 21796441 10.1007/s00792-011-0388-x

[33] Chakrabartty A, Hew CL (1991) The effect of enhanced alpha-helicity on the activity of a winter flounder antifreeze polypeptide. Eur J Biochem 202: 1057–1063. 1765066 10.1111/j.1432-1033.1991.tb16470.x

[34] Haymet ADJ, Ward LG, Harding MM (1999) Winter Flounder “Antifreeze” Proteins: Synthesis and Ice Growth Inhibition of Analogues that Probe the Relative Importance of Hydrophobic and Hydrogen-Bonding Interactions. Journal of the American Chemical Society 121: 941–948.

[35] Knight CA, Hallett J, DeVries AL (1988) Solute effects on ice recrystallization: an assessment technique. Cryobiology 25: 55–60. 3349811 10.1016/0011-2240(88)90020-x

[36] Davies PL, Baardsnes J, Kuiper MJ, Walker VK (2002) Structure and function of antifreeze proteins. Philos Trans R Soc Lond B Biol Sci 357: 927–935. 12171656 10.1098/rstb.2002.1081 PMC1692999

[37] Fairley K, Westman BJ, Pham LH, Haymet AD, Harding MM, et al (2002) Type I shorthorn sculpin antifreeze protein: recombinant synthesis, solution conformation, and ice growth inhibition studies. J Biol Chem 277: 24073–24080. 11940576 10.1074/jbc.M200307200

[38] Knight CA, Driggers E, DeVries AL (1993) Adsorption to ice of fish antifreeze glycopeptides 7 and 8. Biophys J 64: 252–259. 8431545 10.1016/S0006-3495(93)81361-4 PMC1262321

[39] Zhang W, Laursen RA (1998) Structure-function relationships in a type I antifreeze polypeptide. The role of threonine methyl and hydroxyl groups in antifreeze activity. J Biol Chem 273: 34806–34812. 9857006 10.1074/jbc.273.52.34806

[40] Wuthrich K (1986) NMR of Protein and Nucleic Acids. New York: John Wiley & Sons.

[41] Wishart DS, Bigam CG, Holm A, Hodges RS, Sykes BD (1995) ^1^H, ^13^C and^ 15^N random coil NMR chemical shifts of the common amino acids. I. Investigations of nearest-neighbor effects. 10.1007/BF00227471 7881273

[42] Chou PY, Fasman GD (1978) Empirical predictions of protein conformation. Annu Rev Biochem 47: 251–276. 354496 10.1146/annurev.bi.47.070178.001343

[43] Bredenbeck J, Helbing J, Kumita JR, Woolley GA, Hamm P (2005) Alpha-helix formation in a photoswitchable peptide tracked from picoseconds to microseconds by time-resolved IR spectroscopy. Proc Natl Acad Sci U S A 102: 2379–2384. 15699340 10.1073/pnas.0406948102 PMC548979

[44] Wishart DS, Sykes BD, Richards FM (1991) Relationship between nuclear magnetic resonance chemical shift and protein secondary structure. J Mol Biol 222: 311–333. 1960729 10.1016/0022-2836(91)90214-q

[45] Hoshino T, Kiriaki M, Ohgiya S, Fujiwara M, Kondo H, et al (2003) Antifreeze proteins from snow mold fungi. Can J Bot 81: 1175–1181.

[46] Gilbert JA, Hill PJ, Dodd CE, Laybourn-Parry J (2004) Demonstration of antifreeze protein activity in Antarctic lake bacteria. Microbiology 150: 171–180. 14702410 10.1099/mic.0.26610-0

[47] Lee JH, Park AK, Do H, Park KS, Moh SH, et al. (in press) Structural basis for the antifreeze activity of an ice-binding protein from an Arctic yeast. J Biol Chem.

[48] Chakrabartty A, Ananthanarayanan VS, Hew CL (1989) Structure-function relationships in a winter flounder antifreeze polypeptide. I. Stabilization of an alpha-helical antifreeze polypeptide by charged-group and hydrophobic interactions. J Biol Chem 264: 11307–11312. 2738067

[49] Chakrabartty A, Yang DS, Hew CL (1989) Structure-function relationship in a winter flounder antifreeze polypeptide. II. Alteration of the component growth rates of ice by synthetic antifreeze polypeptides. J Biol Chem 264: 11313–11316. 2738068

[50] Houston ME Jr, Chao H, Hodges RS, Sykes BD, Kay CM, et al (1998) Binding of an oligopeptide to a specific plane of ice. J Biol Chem 273: 11714–11718. 9565593 10.1074/jbc.273.19.11714

[51] Hall DG, Lips A (1999) Phenomenology and Mechanism of Antifreeze Peptide Activity. Langmuir 15: 1905–1912.

[52] Nutt DR, Smith JC (2008) Dual function of the hydration layer around an antifreeze protein revealed by atomistic molecular dynamics simulations. J Am Chem Soc 130: 13066–13073. 18774821 10.1021/ja8034027

[53] Epand RM, Vogel HJ (1999) Diversity of antimicrobial peptides and their mechanisms of action. Biochim Biophys Acta 1462: 11–28. 10590300 10.1016/s0005-2736(99)00198-4

[54] Arora A, Tamm LK (2001) Biophysical approaches to membrane protein structure determination. Curr Opin Struct Biol 11: 540–547. 11785753 10.1016/s0959-440x(00)00246-3

[55] Bhunia A, Mohanram H, Bhattacharjya S (2012) Structural determinants of the specificity of a membrane binding domain of the scaffold protein Ste5 of budding yeast: Implications in signaling by the scaffold protein in MAPK pathway. Biochim Biophys Acta 1818: 1250–60. 22285780 10.1016/j.bbamem.2012.01.008

[56] Sicheri F, Yang DS (1995) Ice-binding structure and mechanism of an antifreeze protein from winter flounder. Nature 375: 427–431. 7760940 10.1038/375427a0

[57] Liepinsh E, Otting G, Harding MM, Ward LG, Mackay JP, et al (2002) Solution structure of a hydrophobic analogue of the winter flounder antifreeze protein. Eur J Biochem 269: 1259–1266. 11856360 10.1046/j.1432-1033.2002.02766.x

[58] Knight CA, Cheng CC, DeVries AL (1991) Adsorption of alpha-helical antifreeze peptides on specific ice crystal surface planes. Biophys J 59: 409–418. 2009357 10.1016/S0006-3495(91)82234-2 PMC1281157

[59] Wen D, Laursen RA (1992) Structure-function relationships in an antifreeze polypeptide. The role of neutral, polar amino acids. J Biol Chem 267: 14102–14108. 1629210

[60] Brooke-Taylor CA, Grant GH, Elcock AH, Graham Richards W (1996) Mechanism of action of antifreeze polypeptide HPLC6 in solution: analysis of solvent behaviour by molecular dynamics. Chem Phys 204: 251–261.

[61] Altschul SF, Madden TL, Schaffer AA, Zhang J, Zhang Z, et al (1997) Gapped BLAST and PSI-BLAST: a new generation of protein database search programs. Nucleic Acids Res 25: 3389–3402. 9254694 10.1093/nar/25.17.3389 PMC146917

[62] Altschul SF, Koonin EV (1998) Iterated profile searches with PSI-BLAST–a tool for discovery in protein databases. Trends Biochem Sci 23: 444–447. 9852764 10.1016/s0968-0004(98)01298-5

[63] Thompson JD, Gibson TJ, Higgins DG (2002) Multiple sequence alignment using ClustalW and ClustalX. Curr Protoc Bioinformatics Chapter 2: Unit 2 3. 10.1002/0471250953.bi0203s00 18792934

[64] Bowie JU, Luthy R, Eisenberg D (1991) A method to identify protein sequences that fold into a known three-dimensional structure. Science 253: 164–170. 1853201 10.1126/science.1853201

[65] Bonneau R, Baker D (2001) Ab initio protein structure prediction: progress and prospects. Annu Rev Biophys Biomol Struct 30: 173–189. 11340057 10.1146/annurev.biophys.30.1.173

[66] Jones DT (1999) GenTHREADER: an efficient and reliable protein fold recognition method for genomic sequences. J Mol Biol 287: 797–815. 10191147 10.1006/jmbi.1999.2583

[67] Kelley LA, MacCallum RM, Sternberg MJ (2000) Enhanced genome annotation using structural profiles in the program 3D-PSSM. J Mol Biol 299: 499–520. 10860755 10.1006/jmbi.2000.3741

[68] Shi J, Blundell TL, Mizuguchi K (2001) FUGUE: sequence-structure homology recognition using environment-specific substitution tables and structure-dependent gap penalties. J Mol Biol 310: 243–257. 11419950 10.1006/jmbi.2001.4762

[69] Stott K, Stonehouse J, Keeler J, Hwang T-L, Shaka AJ (1995) Excitation sculpting in high-resolution nuclear magnetic resonance: application to selective NOE experiments. J Am Chem Soc 117: 4199–4200.

[70] Goddard TD, Keller DG SPARKY 3. San Francisco: University of California.

[71] Cornilescu G, Delaglio F, Bax A (1999) Protein backbone angle restraints from searching a database for chemical shift and sequence homology. J Biomol NMR 13: 289–302. 10212987 10.1023/a:1008392405740

[72] Nanga RP, Brender JR, Xu J, Hartman K, Subramanian V, et al (2009) Three-dimensional structure and orientation of rat islet amyloid polypeptide protein in a membrane environment by solution NMR spectroscopy. J Am Chem Soc 131: 8252–8261. 19456151 10.1021/ja9010095 PMC4163022

[73] Guntert P, Mumenthaler C, Wuthrich K (1997) Torsion angle dynamics for NMR structure calculation with the new program DYANA. J Mol Biol 273: 283–298. 9367762 10.1006/jmbi.1997.1284

[74] Laskowski RA, MacArthur MW, Moss DS, Thornton JM (1993) PROCHECK: a program to check the stereochemical quality of protein structures. J Appl Cryst 26: 283–291.

[75] Haris PI, Chapman D (1995) The conformational analysis of peptides using Fourier transform IR spectroscopy. Biopolymers 37: 251–263. 7540054 10.1002/bip.360370404

[76] Brooks BR, Bruccoleri RE, Olafson BD, States DJ, Swaminathan S, et al (1983) CHARMM: A program for macromolecular energy, minimization, and dynamics calculations. J Comput Chem 4: 187–217.

[77] Foloppe N, MacKerell JAD (2000) All-atom empirical force field for nucleic acids: I. Parameter optimization based on small molecule and condensed phase macromolecular target data. J Comput Chem 21: 86–104.

[78] Jorgensen W, Chandrasekhar J, Madura J, Impey R, Klein M (1983) Comparison of simple potential functions for simulating liquid water. J Chem Phys 79: 926–935.

[79] Ryckaert J-P, Ciccotti G, Berendsen H (1977) Numerical integration of the cartesian equations of motion of a system with constraints: molecular dynamics of n-alkanes. J Comput Phys 23: 327–341.

[80] Ewald PP (1921) Die Berechnung optischer und elektrostatischer Gitterpotentiale. Ann Phys 369: 253–287.

[81] Baker NA, Sept D, Joseph S, Holst MJ, McCammon JA (2001) Electrostatics of nanosystems: application to microtubules and the ribosome. Proc Natl Acad Sci U S A 98: 10037–10041. 11517324 10.1073/pnas.181342398 PMC56910

[82] Ritchie DW, Kozakov D, Vajda S (2008) Accelerating and focusing protein-protein docking correlations using multi-dimensional rotational FFT generating functions. Bioinformatics 24: 1865–1873. 18591193 10.1093/bioinformatics/btn334 PMC2732220

