# Supplementary figures and images for: Solution Structures, Dynamics, and Ice Growth Inhibitory Activity of Peptide Fragments Derived from an Antarctic Yeast Protein
Source: PLoS One. 2012 Nov 28;7(11):e49788. doi: 10.1371/journal.pone.0049788 (PMC3509122; doi:10.1371/journal.pone.0049788)

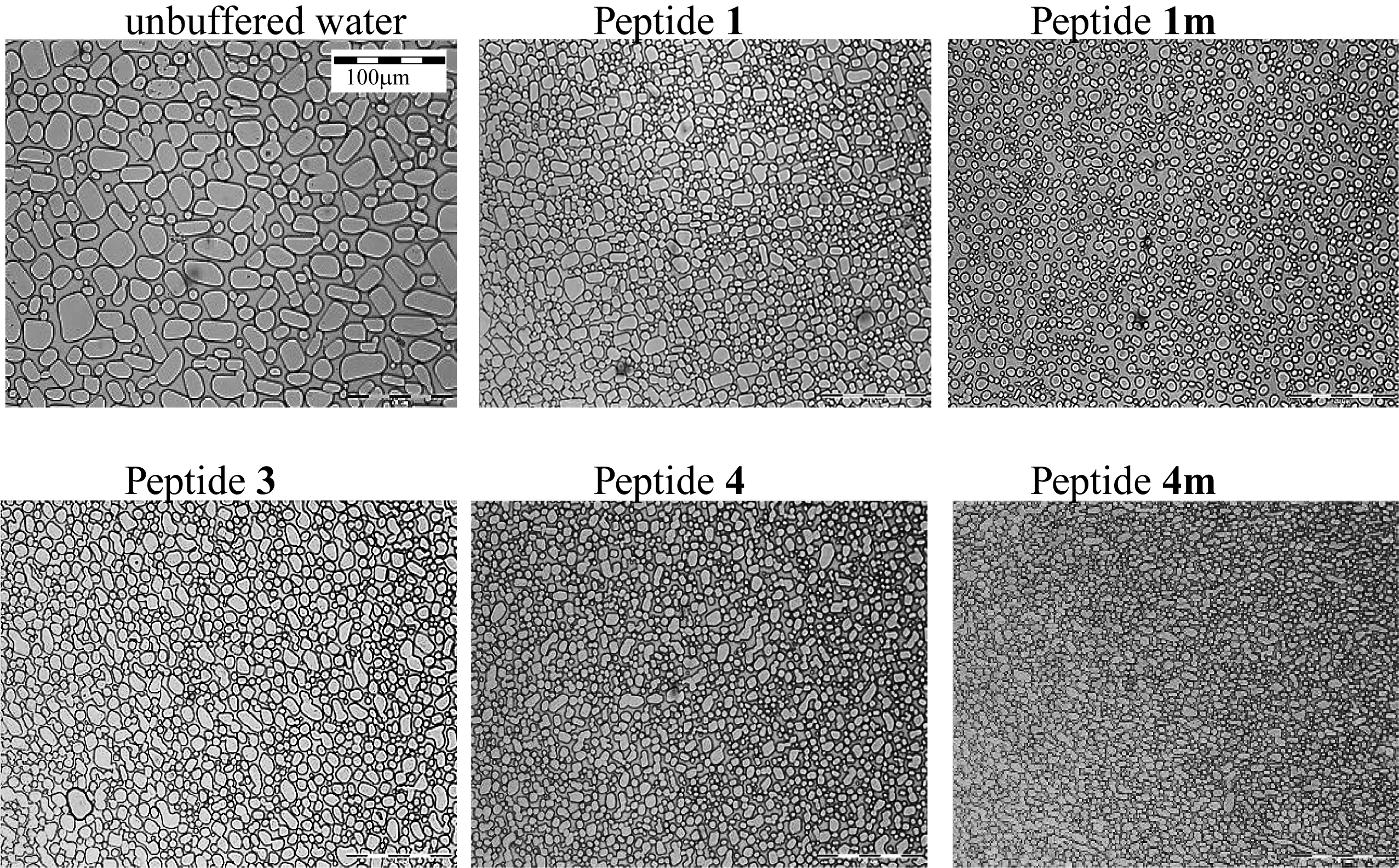

Supplement: Figure S1 — Ice re-crystallization inhibition (IRI) assay results. The growth of ice crystal in the presence of 1 mM peptide was observed for 3 h at the temperature of −6°C. (A) Unbuffered water solution (pH 5.0) without peptide, (B) peptide 1, (C) peptide 1 m, (D) peptide 3, (E) peptide 4, (F) peptide 4 m. The segmented bar represents 100 µm. (TIF) [file pone.0049788.s001.tif]

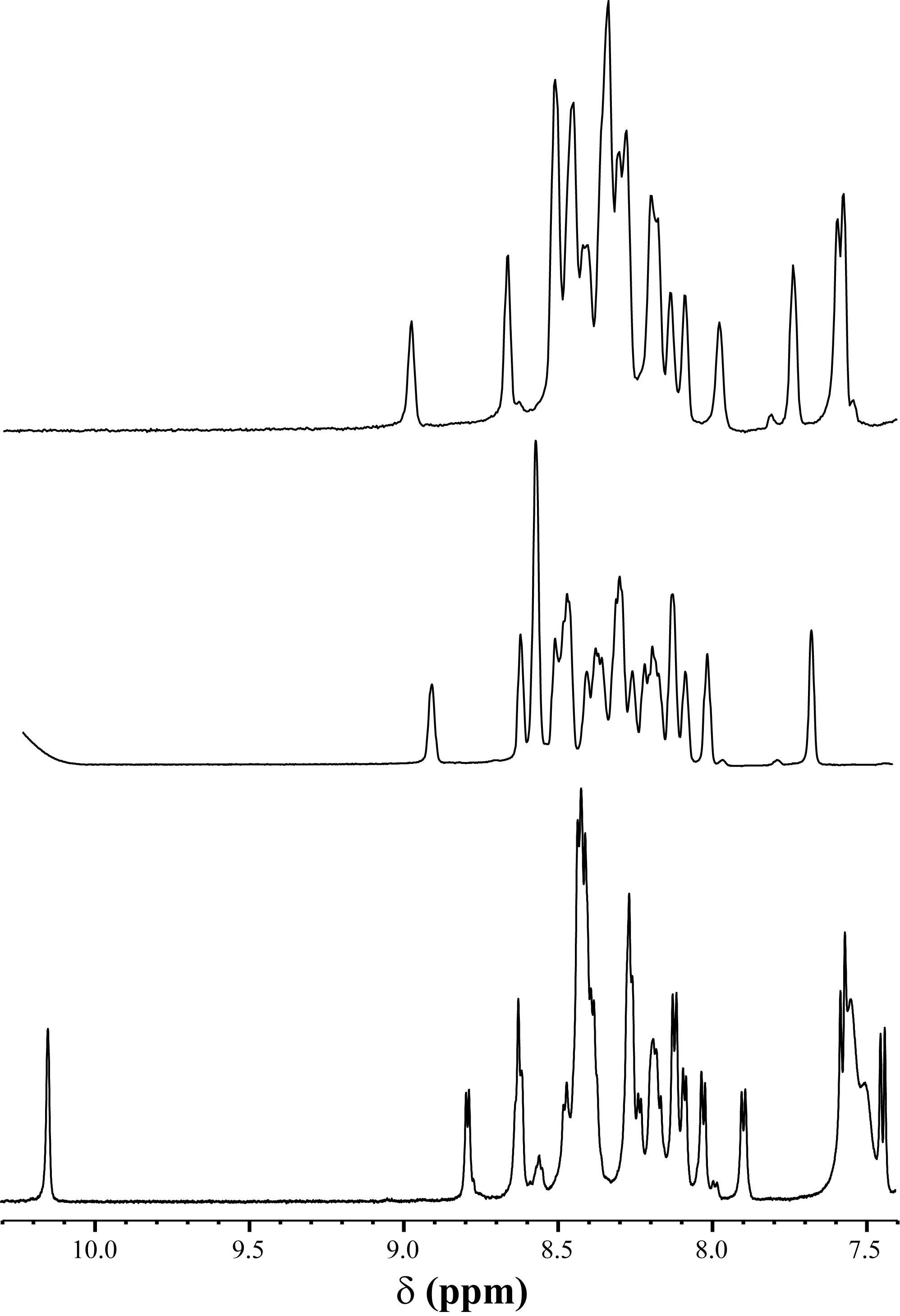

Supplement: Figure S2 — Low field region of the one-dimensional proton NMR spectra of peptide 1 m (top), peptide 3 (middle) and peptide 4 m (below). (TIF) [file pone.0049788.s002.tif]

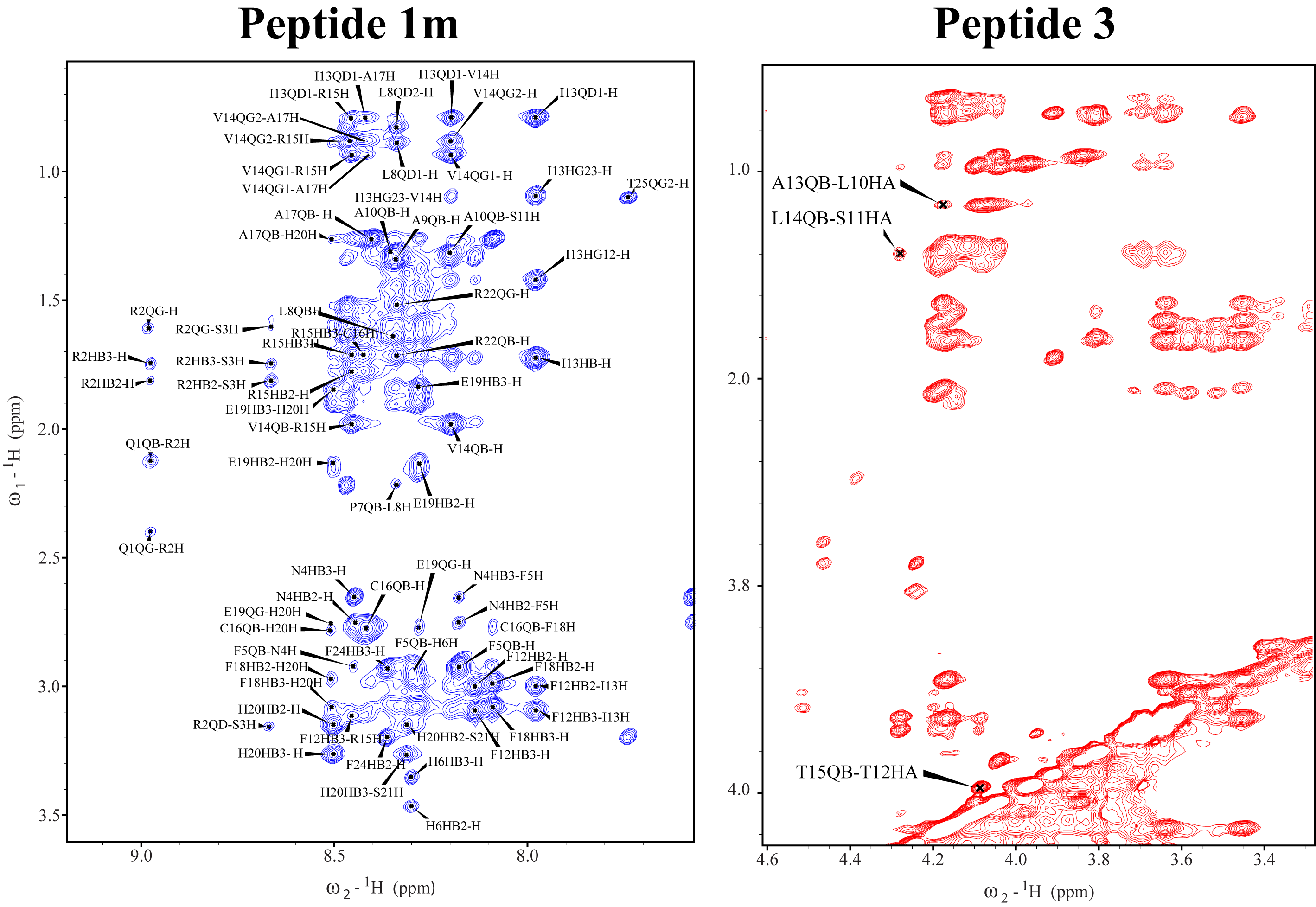

Supplement: Figure S3 — Selected region of two-dimensional 1H-1H NOESY spectra of peptide 1 m (left panel) and peptide 3 (right panel). (TIF) [file pone.0049788.s003.tif]

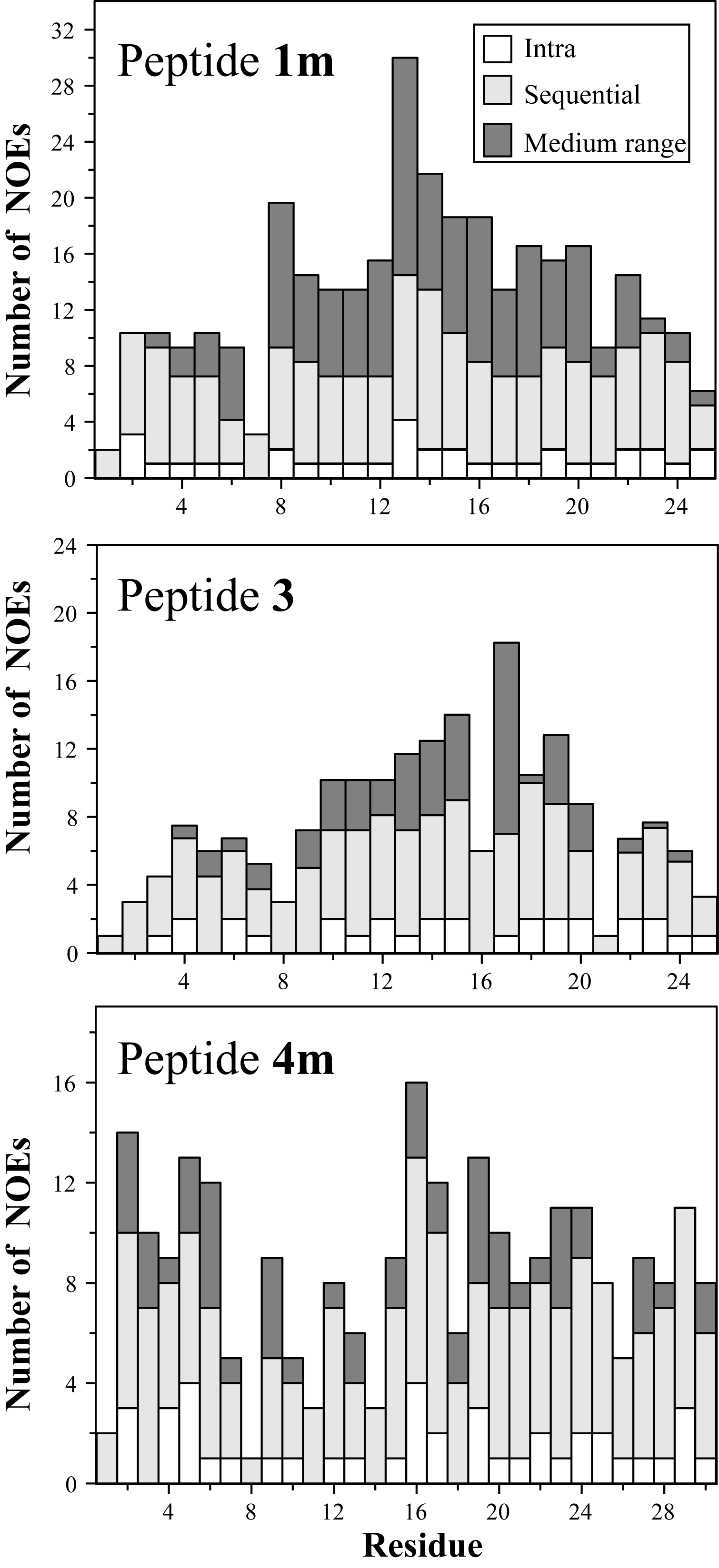

Supplement: Figure S4 — Bar diagram showing the NOE contacts for each residue of peptide 1 m, peptide 3, and peptide 4 m. (TIF) [file pone.0049788.s004.tif]

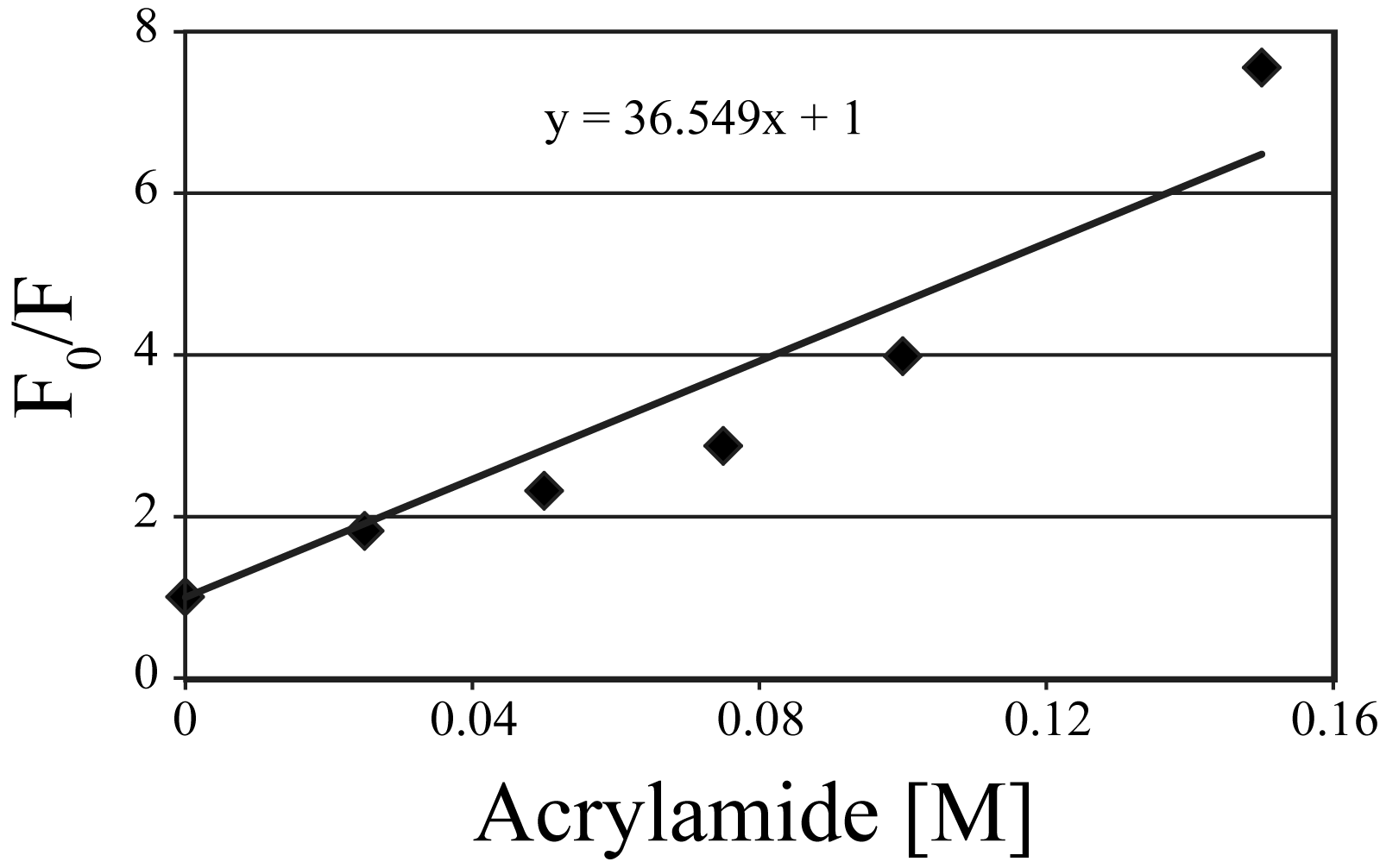

Supplement: Figure S5 — Fluorescence quenching of peptide 4 m by acrylamide in water, pH 5.0. The Stern-Volmer constant (Ksv) for the peptide is 36.55 M−1. (TIF) [file pone.0049788.s005.tif]

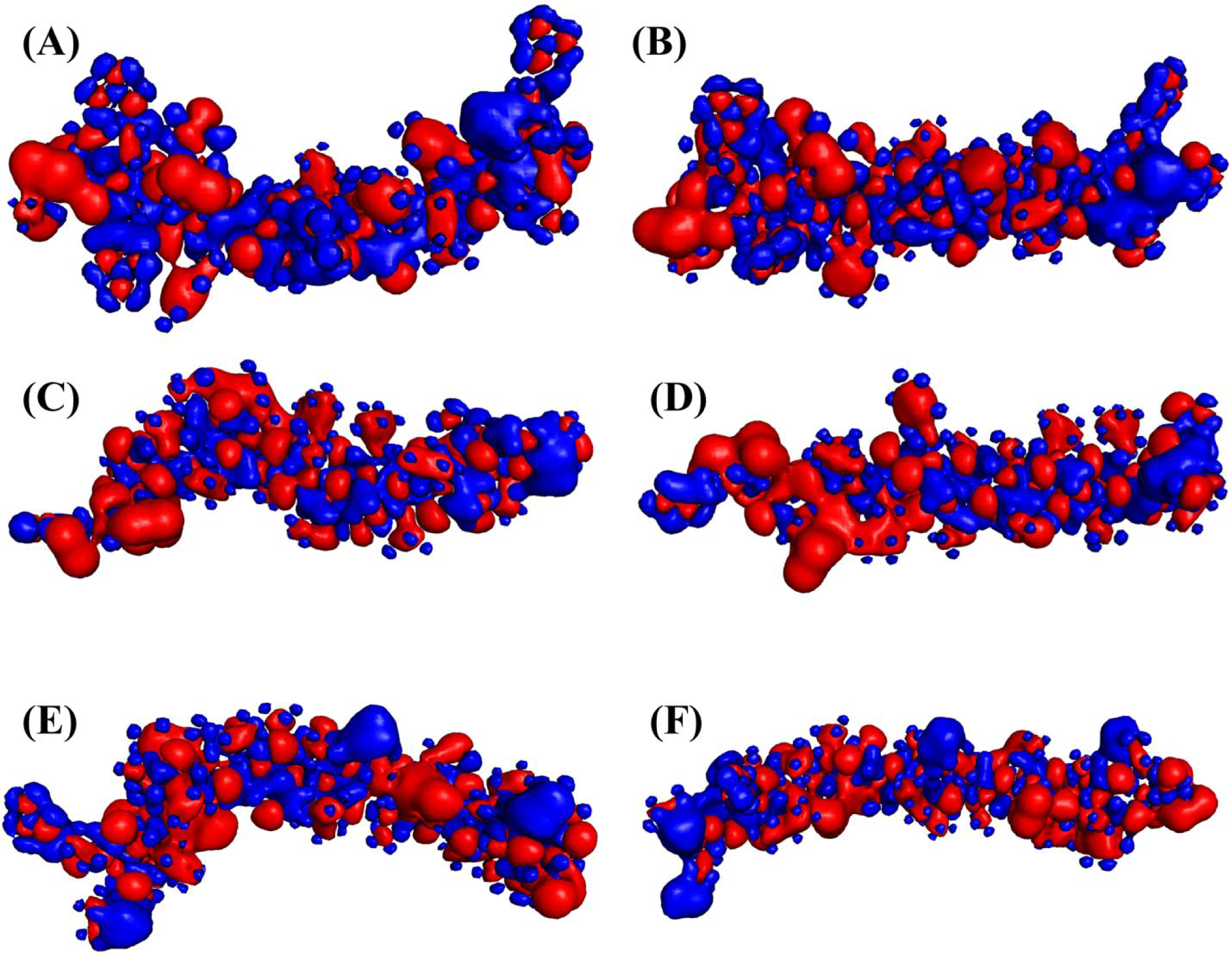

Supplement: Figure S6 — APBS calculation per residues of peptide 1 m (A) NMR structure and (B) after 1.2 ns MD; Peptide 3 (C) NMR structure and (D) after 1.2 ns MD; Peptide 4 m (E) NMR structure and (F) after 1.2 ns MD. Blue color indicates the negatively charged amino acid residue whereas the positively charged residues are marked by blue color. (TIF) [file pone.0049788.s006.tif]
